# Monocytes differentiated into macrophages and dendritic cells in the presence of human IFN-*λ*3 or IFN-*λ*4 show distinct phenotypes

**DOI:** 10.1002/JLB.3A0120-001RRR

**Published:** 2020-11-17

**Authors:** Manjarika De, Anand Bhushan, Sreedhar Chinnaswamy

**Affiliations:** National Institute of Biomedical Genomics, Kalyani,West Bengal, India

**Keywords:** IFN-*λ*3, IFN-*λ*4, immunomodulation, interferon lambda, macrophage polarization, rs12979860, rs368234815

## Abstract

Human IFN-*λ*4 is expressed by only a subset of individuals who possess the ΔG variant allele at the dinucleotide polymorphism rs368234815. Recent genetic studies have shown an association between rs368234815 and different infectious and inflammatory disorders. It is not known if IFN-*λ*4 has immunomodulatory activity. The expression of another type III IFN, IFN-*λ*3, is also controlled by genetic polymorphisms that are strongly linked to rs368234815. Therefore, it is of interest to compare these two IFNs for their effects on immune cells. Herein, using THP-1 cells, it was confirmed that IFN-*λ*4 could affect the differentiation status of macrophage-like cells and dendritic cells (DCs). The global gene expression changes induced by IFN-*λ*4 were also characterized in in vitro generated primary macrophages. Next, human PBMC-derived CD14^+^ mono-cytes were used to obtain M1 and M2 macrophages and DCs in the presence of IFN-*λ*3 or IFN-*λ*4. These DCs were cocultured with CD4^+^ Th cells derived from allogenic donors and their in vitro cytokine responses were measured. The specific activity of recombinant IFN-*λ*4 was much lower than that of IFN-*λ*3, as shown by induction of IFN-stimulated genes. M1 macrophages differentiated in the presence of IFN-*λ*4 showed higher IL-10 secretion than those differentiated in IFN-*λ*3. Coculture experiments suggested that IFN-*λ*4 could confer a Th2-biased phenotype to allogenic Th cells, wherein IFN-*λ*3, under similar circumstances, did not induce a significant bias toward either a Th1 or Th2 phenotype. This study shows for the first time that IFN-*λ*4 may influence immune responses by immunomodulation.

## 1 Introduction

IFN-*λ*4 was unexpectedly discovered during follow-up investigations conducted post-genome-wide association studies that were performed to identify host genetic factors responsible for differential outcomes to therapy against chronic hepatitis C virus (HCV) infections.^1^ IFN-*λ*4 expression is genetically regulated by the dinucleotide DNA polymorphism rs368234815 (TT/ΔG), with individuals harboring the ΔG allele being the only ones able to express the full-length functional IFN-*λ*4.^1^ Approximately 95% of the African population, as well as 50% and 10% of the European and Asian populations respectively, have the potential to express IFN-*λ*4.^1^ RNA viral infection induces IFN-*λ*4 secretion, which in turn can inhibit several viruses in in vitro model systems; however, paradoxically, its expression in vivo is detrimental for HCV clearance in humans.^1–6^ Although IFN-*λ*4 is poorly secreted from cells that express it, it is highly potent in vitro even at low concentrations.^7^ IFN-*λ*4 is only 29% identical to IFN-*λ*3, which is its closest relative in the type III IFN family.^1,8,9^ IFN-*λ*1, another member of the type III IFN family, is known to have immunomodulatory functions and to induce T helper (Th)-1 response,^10,11^ and a very recent report also suggests that IFN-*λ*3 has immunomodulatory activity.^12^ Recent genetic studies have shown an association between IFN-*λ* locus genetic variants, including rs368234815, and various inflammatory disorders^13^ and infections other than HCV.^8,9,14^ Inflammatory diseases, including asthma,^15,16^ chronic obstructive pulmonary disease,^17^ systemic lupus erythematosus,^18^ nonalcoholic fatty liver disease,^19^ liver fibrosis,^20,21^ pulmonary fibrosis,^22^ among others, are known to be influenced by the IFN-*λ* locus genetic polymorphisms. Moreover, a number of infectious diseases other than HCV infections have been associated with the IFN-*λ* locus genetic variants,^7^ including cytomegalovirus infections,^23^ the human immunodeficiency viruses,^24^ respiratory viruses,^25,26^ gastrointestinal viruses,^27^ and even malarial infections.^27^ In addition, some studies have also reported an association between the IFN-*λ* locusgenetic variants with cancers such as prostate cancer^28,29^ and mucinous ovarian carcinoma.^30^ However, the genetic variants that could control IFN-*λ*3 expression and rs368234815 are in strong linkage disequilibrium (LD). Therefore, it is not clear whether IFN-*λ*3 or IFN-*λ*4 is behind these genetic associations^13,14,31^ and is important to clarify their potential roles beyond their antiviral functions. Specifically, it is of interest to understand whether IFN-*λ*3 and IFN-*λ*4 can modulate the properties of immune cells so that they can alter the development of immune cells and influence immune responses.

All four members of the type III IFN family (IFN-*λ*1–4) interact with a heterodimeric receptor IFN-LR1 (or IL28-RA) and IL-10 receptor 2 (IL-10R2) to activate the JAK-STAT pathway and induce expression of IFN-stimulated genes (ISGs).^32^ The receptors for IFN-*λ*s are not ubiquitously expressed and only some immune cells are known to respond to them,^33^ including myeloid and plasmacytoid dendritic cells (DCs),^10,11,34^ B cells,^35^ and neutrophils.^36^ Several previous reports showed that monocytes do not respond to IFN-*λ*s. Doyle et al., while studying HCV, checked for the expression of IFN-LR1 and IL-10R2 in different blood cells,^37^ finding that IFN-LR1 was not expressed in B cells, monocytes, and T cells, although IL-10R2 was highly expressed in these cells.^37^ In the same year, Mennechet and Uze^38^ reported that IFN-LR1 mRNA was expressed in blood DCs and monocytes treated with GM-CSF with or without IL-4 or IFN-*β* (i.e., monocytederived DCs [MoDC]). Moreover, they further showed that IFN-*λ*1-treated DCs were able to induce T regulatory (Treg) cells.^38^ Gallagher and colleagues, however, could see a response from PBMCs to IFN-*λ* 1 stimulus,^10,11^ as well as from MoDCs as measured by their ability to secrete IL-12 and also based on their effect on Th cells.^10^ In another report, isolated and cultured monocytes, but not B (CD19^+^) or T (CD3^+^) cells, were shown to secrete IL-6 in response to IFN-*λ*1.^39^ While characterizing different targets of IFN-*λ*s, Witte et al. found that B and T cells, but not monocytes, could express IFN-LR1; however, only B cells, and not monocytes, T cells, or NK cells, showed STAT1 activation upon treatment with IFN-*λ*.^33^ In 2009, Gallagher and colleagues showed that plasmacytoid DCs and CD4^+^ Th cells can respond to IFN-*λ*s.^34,40^ Dickensheets et al. confirmed that IFN-*α*, but not IFN-*λ*1, was able to signal in primary blood-derived monocytes or lymphocytes.^41^ Moreover, Liu et al.^42^ showed that monocyte-derived macrophages (MDMs), but not monocytes themselves or MoDCs, expressed IL-28RA,^42^ contrary to observations on MoDCs by Mennechet and Uze.^38^ Recently, Freeman et al.^43^ evaluated IL-28RA expression and STAT1 signaling upon IFN-*α* and IFN-*λ* treatment in hepatocytes and PBMCs and found that the pathway was intact in hepatocytes whereas it was not in PBMCs.^43^ Boonstra and colleagues confirmed that monocytes themselves do not respond to IFN-*λ*, but GM-CSF treatment induces IFN-LR1 expression, leading to a response.^44^ More recently, Read et al. confirmed that blood-derived monocytes do not express IFNLR1; however, upon differentiation into macrophages by macrophage CSF (M-CSF) or GM-CSF, they gain IFN-*λ*3 response by induction of IFN-LR1 expression, overall showing that IFN-*λ*3 can modulate macrophage responses.^12^

To date, no studies have explored the immunomodulatory function of IFN-*λ*4, even though a number of reports have addressed its antiviral functions.^2–5^ Macrophages are one of the first immune cells to respond to an insult, pathogenic or sterile. They are also intricately involved in development and homeostasis. DCs are the link between the innate and adaptive immune responses and are also required to maintain peripheral tolerance.^45^ Because IFN-*λ*4 is expressed in only a subset of individuals, it is necessary to understand its influence on these two important immune cell subsets that may impact on the several inflammatory and infectious disorders to which the IFN-*λ* locus is associated with.^8,9,13,14^ Because the occurrence of the alleles that promote the expression of IFN-*λ*3^46,47^ and that which gives rise to IFN-*λ*4 can be mutually exclusive due to strong LD in the majority of human populations,^1,8,9,13,14^ it would be of interest to evaluate the differential effects of IFN-*λ*3 and IFN-*λ*4 on immune cells. The present study aimed to address all these unanswered questions.

## 2 Materials and Methods

### 2.1 Cell culture and differentiation

Human monocytic cell lines THP-1 or U937 (purchased from the American Type Culture Collection [ATCC], Manassas, VA, USA) were maintained in RPMI-1640 medium, 10% FBS, and penicillin/streptomycin (all from Gibco, Waltham, MA, USA) at 37° and 5% CO_2_. A549 cells (ATCC) were maintained in DMEM supplemented with 10% FBS and penicillin/streptomycin (Gibco).

Human recombinant IFN-*λ*4 (catalog #9165-IF; carrier-free form) and IFN-*λ*3 (catalog #5259-IL/CF), containing <0.1 EU/*μ*g of endotoxin, were purchased from R&D Systems (Minneapolis, MN, USA). To generate M1-activated macrophage-like cells, THP-1 cells (5 × 10^5^/ml) were seeded along with 100 nM PMA (Sigma-Aldrich, St. Louis, MO, USA) in the absence or presence of 2 or 6 *μ*g/ml of IFN-*λ*4 (pretreatment strategy), and incubated for 48 h. Afterward, the media was removed and any cells present in the media were pelleted and seeded back into the same wells along with fresh medium containing 1 *μ*g/ml of LPS (Sigma-Aldrich) with or without 50 ng/ml recombinant IFN-*γ* (R&D Systems) for M1 polarization. In posttreatment experiments, IFN-*λ*4 protein (concentration ranging between 1 and 6 *μ*g/ml) was added or not to THP-1 cells after PMA treatment for 48 h along with LPS. After LPS treatment (in both strategies), cells were incubated for 24 h and cell-free media were collected for ELISA and the cells were used for quantitative polymerase chain reaction (qPCR) or flow cytometry analyses. To generate M2-activated macrophage-like cells, THP-1 cells (5 × 10^5^/ml) were seeded along with 300 nM PMA in the absence or presence of IFN-*λ*4 (6 *μ*g/ml). After 6 h, 100 ng/ml of human recombinant M-CSF, IL-4, and IL-10 (all from R&D Systems) were added to the existing media. After 48 h, cells were either processed for flow cytometry or harvested for qPCR experiments. To generate DCs from THP-1 cells, 5 × 10^5^/ml cells were seeded along with 100 ng/ml of human recombinant GM-CSF and IL-4 (R&D Systems) in the presence or absence of IFN-*λ*4 at 6 *μ*g/ml (pretreatment strategy). On day 3, the medium was removed and any cells in the media were pelleted and seeded back to the respective wells along with fresh cytokines with or without IFN-*λ*4. On day 5, the medium was replaced with fresh medium containing 1 *μ*g/ml of LPS. After 24 h of further incubation, cell-free supernatants were collected for ELISA and the cells were processed for flow cytometry or qPCR experiments.

For generation of MDMs and MoDCs: 10 × 10^6^ CD14^+^ monocytes from five donors were purchased from PromoCell (Heidelberg, Germany) (C12909, Lot. 451Z030, 29 yr, male Caucasian; 454Z021, 40 yr, female Caucasian; 449Z030, 31 yr, male Caucasian; 450Z016, 33 yr, male Caucasian; and 452Z017, 41 yr, male Caucasian). The cells were ∼99% homogenous as determined by microscopy. The CD14^+^ cells were initially maintained in the PromoCell mononuclear cell medium for 24 h for revival. In all later steps, they were maintained in RPMI-1640 with 10% FBS and Pen/Strep at 37°C, 5% CO_2_.

For MDM generation, 7.5 × 10^5^ cells/ml were seeded in 24-well plates (0.5 ml/well) and supplemented with GM-CSF (400 U/well) for M1-MDM and M-CSF (50 ng/well) for M2-MDM, in the presence or absence of IFN-*λ*4 (6 *μ*g/ml) or IFN-*λ*3 (50 ng/ml). On days2 and 5, the medium was changed and fresh GM-CSF/M-CSF and IFN-*λ*4 (6 *μ*g/ml) or IFN-*λ*3 (50 ng/ml) were added. On day 6, both M1-MDM and M2-MDM were activated by treatment with LPS (100 ng/ml) for 24 h. Cell-free medium was collected for ELISA and cell pellets were used for RNA isolation. MoDCs were generated by culturing 1.25 × 10^6^ CD14^+^ monocytes/ml in 12-well plates (1 ml/well) with IL-4 (400 U/well), GMCSF (800 U/well) in the presence or absence of IFN-*λ*4 (6 *μ*g/ml) or IFN-*λ*3 (50 ng/ml) for a total of 5 d. On day 3, the media were changed and replenished with fresh cytokines with or without IFN-*λ*4 or IFN-*λ* 3. On day 5, the MoDCs were activated by adding LPS (1 *μ*g/ml) for 24 h.

### 2.2 Surface staining of cells for flow cytometry

IFN-*λ*4-treated and untreated PMA-differentiated THP-1 macrophage-like cells, fresh THP-1 cells, and THP-1-derived DCs, were harvested and incubated with human Fc block (BD Biosciences, San Jose, CA, USA) before staining. The cells were stained with PE Cy7-conjugated anti-human CD80 mAb, APC-conjugated anti-human CD86 mAb, APCH7-conjugated anti-human HLADR mAb, PEconjugated anti-human CD209 mAb, BV421-conjugated anti-human CD163 mAb, PE-conjugated anti-human CD206 mAb, PE-conjugated anti-human T-cell immunoglobulin and mucin-domain containing-3 (TIM-3) mAb, and PE-conjugated IL28 RA polyclonal Ab or isotypematched control mAbs. All antibodies were purchased from BD Biosciences except PE-conjugated IL28 RA Ab and PE-conjugated TIM-3, which were purchased from Biolegend (San Diego, CA, USA). The cells were analyzed using BD Aria Fusion cytometer and the FCS express 6 (De Novo Software, Pasadena, CA, USA).

### 2.3 Western blot and ELISA

To check for the expression of total and activated STAT1 in THP-1 cells, treated and untreated cells were lysed with RIPA buffer and protein estimation was performed using a BCA protein assay kit (Pierce Biotechnology, Waltham, MA, USA). Protein was run on a 10% SDS polyacrylamide gel. Western blot was carried out using anti-STAT1 mAb (Biolegend), anti-pSTAT1 mAb (Biolegend), and beta-actin (Santa Cruz Biotechnology, Dallas, TX, USA) primary antibodies at 1/100 dilution, and goat anti-mouse HRP-conjugated secondary antibody (Millipore, Burlington, MA, USA; 1:1000 dilution). Western blot analysis of human recombinant IFN-*λ*4 run on a 10% SDS-PAGE gel was carried out using a rabbit IFN-*λ*4 mAb at 1/100 dilution (Abcam, Cambridge, United Kingdom) and goat anti-rabbit HRP-conjugated secondary antibody at 1/1000 dilution (Millipore).

Western blot for pSTAT1 in M2-MDM cells: CD14^+^ monocytes from healthy volunteers were isolated using the EasySep Human Monocyte Isolation Kit (negative selection, catalog #19059, StemCell Technologies, Vancouver, Canada). On day 7 of culture (described in Section 2.1), M2-MDM and fresh CD14^+^ monocyte cells isolated from the same healthy donor were treated with IFN-*λ*3 (100 ng/ml). After 30 min, the cells were harvested, washed, lysed with RIPA buffer, and protein estimation was performed using a BCA protein assay kit (Pierce Biotechnology). The proteins were separated on a 10% SDS polyacrylamide gel, and anti-pSTAT1 (Tyr701) mAb (Cell Signaling Technology, Danvers, MA, USA) and beta-actin (Santa Cruz Biotechnology) primary antibodies at 1/1000 dilution and goat anti-mouse HRPconjugated secondary antibody (Millipore; 1:1000 dilution) were used.

Western blot for pSTAT1 from MoDCs: CD14^+^ monocytes from a healthy volunteer were isolated using the EasySep Human Monocyte Isolation Kit (positive selection, catalog #17858, StemCell Technologies). On day 7 of culture (described in Section 2.1), MoDCs were treated with IFN-*λ*4 (6 *μ*g/ml). After 30 min, the cells were harvested, washed, lysed with RIPA buffer, and protein estimation was performed as previously described. The proteins were separated on a 10% SDS polyacrylamide gel, and anti-pSTAT1 (Tyr701) mAb (Cell Signaling Technology) and beta-actin (Santa Cruz Biotechnology) were used as described above.

ELISA was performed to measure secreted TNF-*α*, IL-4, IL-6, IL-1*β*, IFN-*α*, IL-17, IL-10, IL-12, and IL-13 in cell-free supernatants using human DuoSet ELISA kits purchased from R&D Systems and following the manufacturer’s protocols.

### 2.4 Coculture of MoDCs and Th cells

The institutional ethics committee approved this study and informed consent was obtained from all human volunteers donating blood. To isolate CD4^+^ cells from PBMCs for the initial experiments involving IFN-*λ*4, eight healthy volunteers (median age 27 yr; 4 males and 4 females) were recruited. For later experiments, 20 healthy volunteers were recruited, among whom 10 were from a younger age group (median age 28 yr; 3 males and 7 females) and the other 10 belonged to an older age group (median age 43 yr; 6 males and 4 females). The younger donors also belonged to a higher socioeconomic status than the older donors based on their profession and place of residence. PBMCs were first isolated from healthy donors using the Ficoll-Paque (Sigma-Aldrich) method^48^ followed by CD4^+^ Th cell separation using the Easy SepTM Human CD4^+^Th cell isolation kit (negative selection, catalog #17952, StemCell Technologies). Activated MoDCs were generated as described in Section 2.1 from CD14^+^ monocytes, were washed and seeded into 12-well plates along with CD4^+^Th cells in 2 ml of media. The ratio of coculture was maintained at 1:10 (5 × 10^4^ MoDCs: 5 × 10^5^ CD4^+^Th cells). On days 3 and 6, 1 ml of medium was replaced with fresh medium in the presence of human recombinant IL2 (10 U/ml, R&D Systems) for the expansion of Th cells. On day 9, cultured cells were harvested and washed three times. Cells were then collected and resuspended in 2 ml media containing PMA (10 ng/ml) and ionomycin (1 *μ*g/ml, MP Biomedicals, Santa Ana, CA, USA), transferred onto a new 12-well plate, and cultured for further 18 h. Supernatants were harvested and frozen for later cytokine analysis by ELISA.

### 2.5 Quantitative PCR

Total RNA was isolated from cell pellets of activated THP-1-derived macrophage-like cells and DCs, A549 cells, activated MDMs (generated from CD14^+^ cells purchased from PromoCell or from CD14^+^ cells purified from a healthy donor), and MoDCs (purified from CD14^+^ cells purified from a healthy donor) using the RNeasy kit (Qiagen, Hilden, Germany). Purified RNA (500 ng) was converted to cDNA using the High Capacity cDNA Reverse Transcription Kit (Applied Biosystems, Waltham, MA, USA) or Verso cDNA Synthesis Kit (Thermo Scientific, Waltham, MA, USA). Gene-specific primers and SYBR Green PCR Master Mix (from Applied Biosystems or Bio-Rad, Hercules, CA, USA) were used. qPCR was performed using either ABI7900HT (Applied Biosystems) or CFX96 Real-Time System (Bio-Rad). *GAPDH* was used as internal control and relative expression was calculated using the 2^−ΔΔCT^ method. The primers used in the study are listed in Supporting Information Table S1.

### 2.6 RNA sequencing (RNA-seq) and bioinformatics

RNA-seq and data analysis were carried out at QuickBiology (Pasadena, CA, USA). RNA integrity was checked using a Bioanalyzer 2100 (Agilent Technologies, Santa Clara, CA, USA) and samples with clean rRNA peaks (RIN > 7) were used for further experiments. Library for RNA-seq was prepared according to KAPA stranded mRNA-Seq poly-A selected kit with a 201–300 bp insert size (KAPA Biosystems, Wilmington, MA, USA) using 250 ng total RNA as input. Final library quality and quantity were analyzed using an Agilent Technologies Bioanalyzer 2100 and Life Technologies Qubit 3.0 Fluorometer (Carlsbad, CA, USA). 150 bp paired-end reads were sequenced on an HiSeq 4000 (Illumina, San Diego, CA, USA).

The reads were first mapped to the latest UCSC Genome Browser transcript set using Bowtie2 version 2.1.0,^49^ and the gene expression level was estimated using RSEM v1.2.15.^50^ Trimmed mean of M-values was used to normalize gene expression and differentially expressed genes (DEGs) were identified using the edgeR software.^51^ Genes showing altered expression with *P* < 0.05 and more than 1.5-fold change were considered differentially expressed. “Goseq” R package was used to perform GO enrichment analysis, and Kobas (http://kobas.cbi.pku.edu.cn/) was used to perform pathway analysis.

### 2.7 Statistical analysis

The mean values of different samples were compared using unpaired or paired 2-tailed or 1-tailed Student’s *t*-test, as indicated in the fig. legends. Comparisons between normalized data were performed using a one-sample *t*-test. *P*-values lower than 0.05 were considered statistically significant.

## 3 Results

In the initial part of the study, a detailed characterization of the modulatory effect of IFN-*λ*4 on macrophages and DCs was performed. In the later part of the study, the effects of IFN-*λ*4 and IFN-*λ*3 on MDMs and MoDCs were compared.

### 3.1 IFN-*λ*4 can signal in PMA-treated THP-1 cells

**Figure 1:**
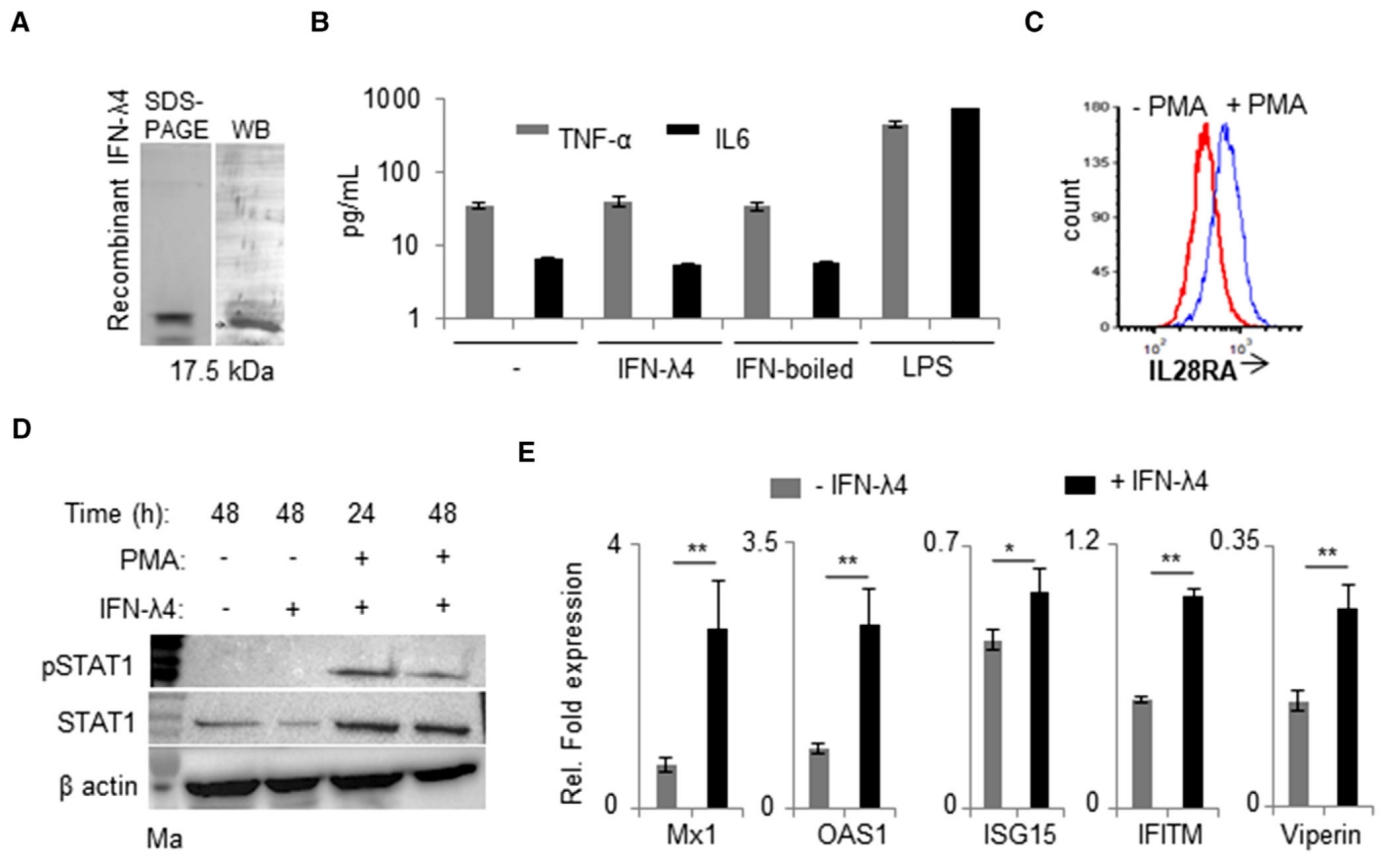
IFN-*λ*4 can signal in monocytes after PMA treatment. (**A**) Western blot analysis of the human recombinant IFN-*λ*4 used in the study. (B) TNF-*α* and IL-6 cytokines secretion was measured by ELISA from cell-free supernatants of THP-1-derived macrophage-like cells treated for 48 h with1 *μ*g/ml IFN-*λ*4, boiled IFN-*λ*4 preparation, or 1 *μ*g/ml of LPS; untreated macrophages served as control; mean and error bars depicting _SD_ from technical replicates are shown. (**C**) THP-1 cells were differentiated into macrophage-like cells by treatment with PMA (100 nM) for 48 h. The cells were removed and compared with fresh untreated THP-1 cells for surface expression of IL28RA using flow cytometry. (**D**) pSTAT1 and STAT1 expression was assessed by Western blot in THP-1 cells treated or not with PMA (100 nM) in the presence or absence of IFN-*λ*4 (1 *μ*g/ml) for 24 and 48 h as indicated. *α*-actin was used as loading control. The presented immunoblot is a representative image of two independent experiments. derived macrophage-like cells treated or not with 5 *μ*g/ml of IFN-*λ*4 for 24 h. The data are representative of two independent experiments showing (**E**) IFN-stimulated genes expression was analyzed by quantitative polymerase chain reaction (qPCR) based on RNA samples extracted from THP-1mean of technical replicates from a single experiment with _SD_ depicted by error bars. *P < 0.05; **P < 0.01

A commercially available IFN-*λ*4 recombinant protein expressed and purified from *Escherichia coli* was used in this study, and the specificity of the protein was confirmed by Western blotting (Fig. 1A). When PMA-treated THP-1 cells were incubated with IFN-*λ*4 for 48 h, no effect was observed on TNF-*α* and IL-6 cytokine secretion (Fig. 1B), suggesting that the recombinant protein was free from any endotoxin contamination. Flow cytometry analysis revealed that PMA-treated THP-1 cells^52^ had increased IFN-LR1 expression (Fig. 1C). Next, the IFN-*λ*4 signaling pathway was evaluated by probing for phosphorylated STAT1 (pSTAT1) in THP-1 cells before and after addition of IFN-*λ*4 and PMA (Fig. 1D). The results showed that the monocytic cell line could not support IFN-*λ*4 signaling; however, PMA treatment for 24 h was sufficient to promote STAT1 phosphorylation, suggesting that IFN-*λ*4 was able to signal in THP-1 cells that were subject to differentiation into macrophage-like cells induced by PMA.^52^ However, PMA treatment also increased the expression of total STAT1 (Fig. 1D). The expression of ISGs was evaluated next in PMA-treated THP-1 cells upon IFN-*λ*4 stimuli (Fig. 1E).

### 3.2 IFN-*λ*4-treated PMA-differentiated THP-1 macrophage-like cells show a subdued M1 phenotype

**Figure 2:**
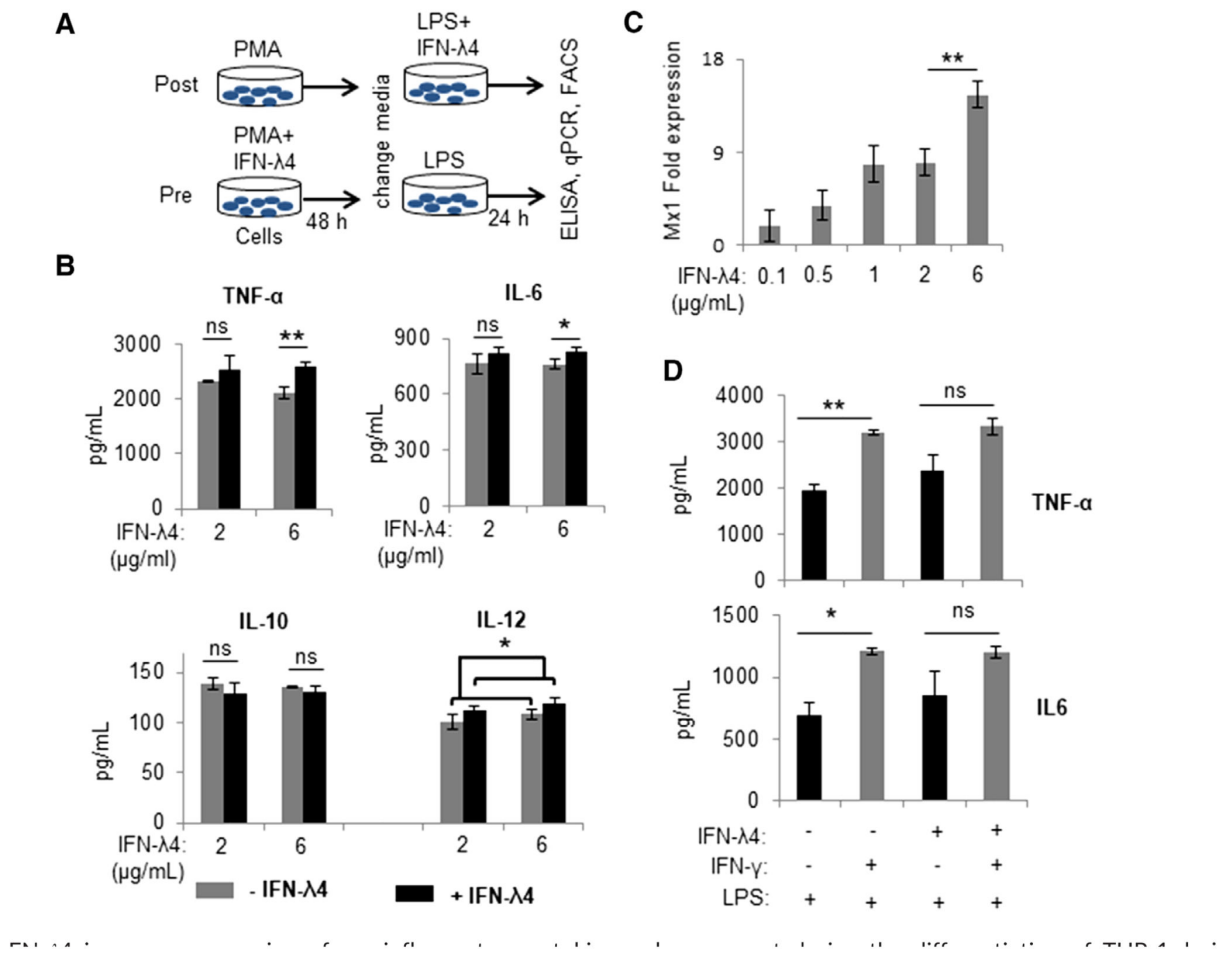
IFN-*λ*4 increases expression of pro-inflammatory cytokines when present during the differentiation of THP-1-derived M1 macrophage-like cells. (**A**) Schematic representation of the two strategies (postand pretreatment) used to examine the effect of IFN-*λ*4 on macrophage-like cells. (**B**) IFN-*λ*4 significantly increased the secretion of TNF-*α*, IL-6, and IL-12 in the pretreatment strategy (at a higher dose) showing the mean and _SD_. (**C**) MX dynamin-like GTPase 1 (*Mx1)* expression in A549 cells treated for 24 h with graded doses of IFN-*λ*4. The data from THP-1-derived macrophage-like cells activated toward an M1 phenotype by LPS treatment. The data are from three biological replicates show the mean of technical replicates with error bars showing the _SD_ (a significant difference, *P* < 0.01, in the expression of *Mx1* between 2 and 6 *μ*g/ml of recombinant IFN-*λ*4 was observed). (**D**) IFN-*λ*4 and IFN-*λ* may activate overlapping pathways. THP-1-derived macrophage-like cells differentiated with ±IFN-*λ*4 were activated to an M1 phenotype by using LPS and ±IFN-*λ*. The data are from three biological replicates showing the mean and _SD_. For (**B**), (**C**), and (**D**) *P < 0.05; **P < 0.01; ns, not significant

The following experiments were designed to assess the effect of IFN-*λ* 4-treated macrophage-like cells derived from THP-1 cells treated with PMA. LPS was used to activate the cells to an M1 phenotype.^52,53^ In order to standardize the protocols, two strategies were followed: (i) posttreatment—differentiation of THP-1 cells was first induced with PMA for 2 d, after which the cells were then activated with LPS in the presence of IFN-*λ*4; and (ii) pretreatment— IFN-*λ*4 was used along with PMA for 2 d to induce differentiation of THP-1 cells, followed by activation with LPS (Fig. 2A). Cytokine production was determined 24 h after LPS treatment in both strategies. In the posttreatment strategy, multiple independent experiments failed to show a consistent and significant effect of IFN-*λ*4 on activated macrophage-like cells in cytokine responses (data not shown). In contrast, in the pretreatment strategy, a significant effect was observed at a higher dose (6 *μ*g/ml) but not at a lower dose (2 *μ*g/ml) of IFN-*λ*4 on the secretion of proinflammatory cytokines, such as TNF-*α*, IL-6, and IL-12 (Fig. 2B). The antiinflammatory cytokine IL-10 did not show such an increase and even showed an opposite trend, suggesting that IFN-*λ*4 was able to enhance the “M1 phenotype”^53^ of the macrophage-like cells in vitro. Certainly, the modulatory effect of IFN-*λ*4 on macrophage-like cells is not as strong as the effect of other IFNs such as IFN-*λ* (see in following text), considering the fact that IFN-*λ*4 is a potent antiviral cytokine.^9^ Such small changes in cytokine levels measured in the in vitro assays may or may not lead to biologically significant outcomes, but their accumulation over time may have relevant consequences for the immune system and its response. Additional experiments in another monocytic cell line, U937, also showed similar results with TNF-*α* stimulus (Supporting Information Fig. S1a). Nevertheless, it is important to note that a high concentration (6 *μ*g/ml) of IFN-*λ*4 was necessary to observe a significant effect on cytokine release. To determine the effect of such a high concentration on ISG expression, increasing IFN-*λ*4 concentrations were tested using A549 cells and MX dynamin-like GTPase 1 (Mx1) expression was measured (Fig. 2C). We saw that Mx1 expression had not reached saturation levels at 2 ug/ml of IFN-*λ*4 in the media. We saw a <2-fold, but statistically significant increase when IFN-*λ*4 concentration was increased to 6 ug/ml in the media. Moreover, no signs of cell distress (in both THP-1 and A549) were seen at high IFN-*λ*4 concentrations. It is known that *E. coli-*expressed IFN-*λ*4 is difficult to refold,^54^ meaning that a higher concentration may be needed to see significant effects. Of note, a commercial preparation of the protein reconstituted from different batches of the lyophilized form was used in these experiments, and the concentrations indicated were prepared as per the manufacturer’s guidelines. Based on these results, the pretreatment strategy was used in subsequent experiments with an IFN-*λ* 4 “effective” concentration of 6 *μ*g/ml in the culture media.

**Figure 3:**
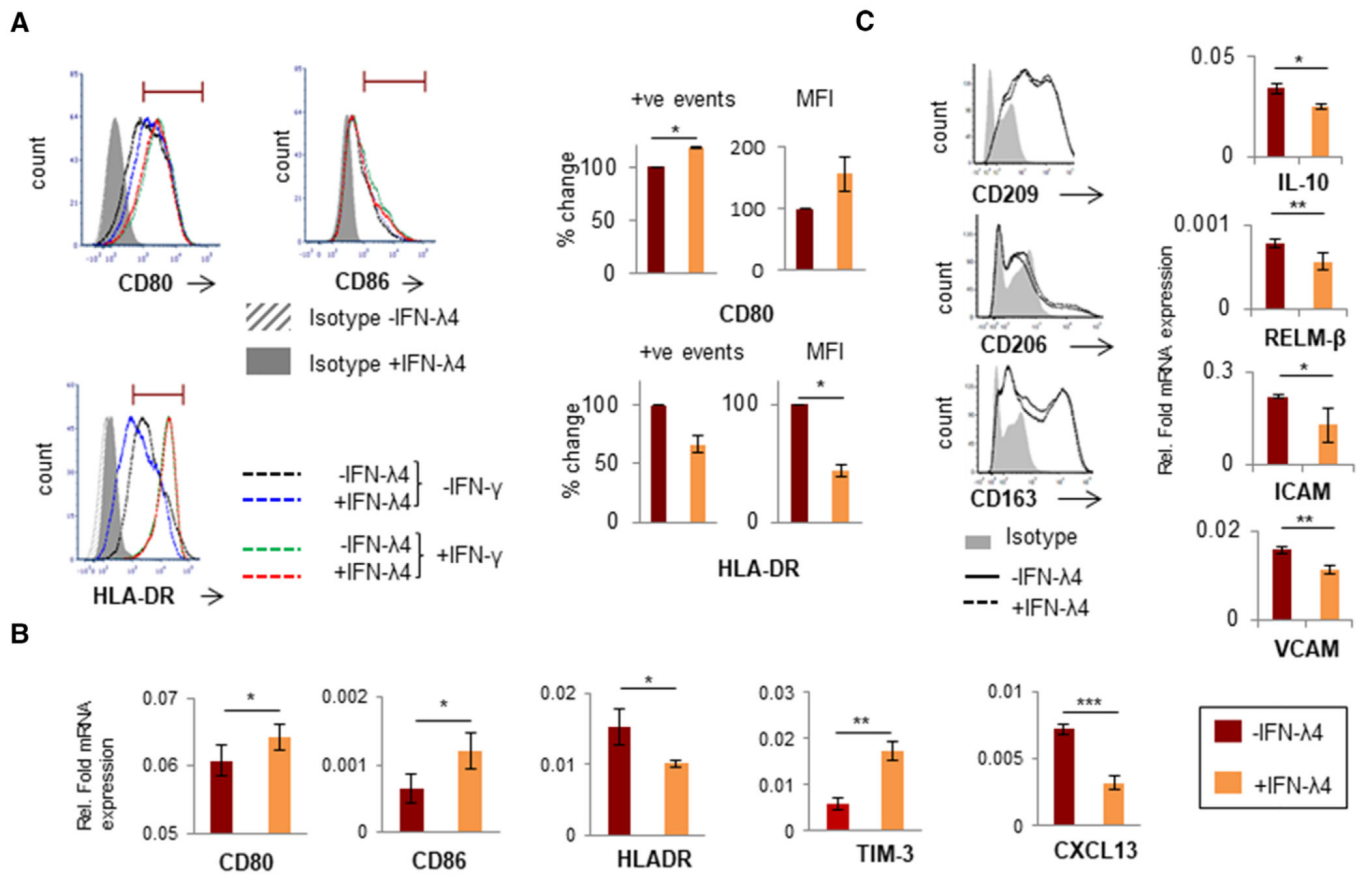
IFN-*λ*4 confers a mixed phenotype on THP-1-derived macrophage-like cells. (**A**) Histogram from one representative experiment showing that IFN-*λ*4-treated macrophage-like cells had increased expression of CD80 but reduced expression of HLA-DR (left) when activated with LPS, whereas IFN-*λ*4 had no effect when activation was carried out with LPS and IFN-*λ*. The graph on the right shows the normalized (for mock IFN-*λ*4 treatment; error bars show _SD_. The significance was calculated using a one-sample *t*-test (*P < 0.05). (**B** and **C**, right) mRNA expression of diftreatment) mean of positive events in the gate shown in the histogram and/or mean fluorescence intensity from two independent experiments with ferent genes from M1-activated (with LPS) (**B**) and M2-activated (with macrophage CSF [M-CSF], IL-4, and IL-10) (**C**) THP-1-derived macrophagereplicate experiments. *P < 0.05; **P < 0.01, ***P < 0.001. (**C**, left) Histogram of CD209-, CD206-, and CD163-positive cells as determined by flow like cells. The data show the mean from technical triplicates with error bars depicting _SD_, and are representative of at least two separate biological cytometry of THP1-derived M2 macrophage-like cells differentiated in the absence or presence of IFN-*λ*4 (6 *μ*g/ml). The data were obtained from a single experiment

Next, IFN-*λ* and LPS were used to activate macrophage-like cells (Fig. 2D). As expected, IFN-*λ* significantly induced the secretion of TNF-*α* and IL-6 compared with LPS alone, but the effect decreased in cells that were differentiated along with IFN-*λ*4, suggesting that both IFNs likely activate overlapping pathways (Fig. 2D). These results suggest that IFN-*λ*4 could promote the differentiation of monocytes to a proinflammatory M1 phenotype.^55^ Flow cytometry and mRNA analysis were performed next to examine the expression of other M1 and M2 macrophage markers (only genes that showed significant expression changes are shown in Fig. 3; all genes tested are shown in Supporting Information Table S1). Although CD80 (a costimulatory molecule and M1 marker) expression was enhanced in cells differentiated in the presence of IFN-*λ*4, a decrease in the expression of the HLADR marker was observed (Fig. 3A and B), whereas IFN-*λ*4 had no effect when macrophage-like cells were activated with LPS and IFN-*λ* (Fig. 3A) similar to results with cytokine expression (Fig. 2D). mRNA expression analysis further showed higher expression of CD80 (and CD86), but a decrease in HLA-DR expression, which is in agreement with flow cytometry results (Fig. 3A and B). Moreover, TIM-3, which is an M2 marker and an important factor required for the scavenging activity of macrophages,^56^ as well as CXCL13, an M1 marker,^57^ were significantly affected by IFN-*λ*4 treatment. TIM-3 expression was elevated at both the mRNA and protein levels (Fig. 3B and Supporting Information Fig. S1b), whereas CXCL13 expression was reduced (Fig. 3B), which is suggestive of a mixed phenotype wherein features of both M1 and M2 macrophages are seen. Afterward, THP-1 cells were differentiated into M2 macrophage-like cells by treatment with PMA (±IFN-*λ*4) for 6 h, followed by replacement with media containing M-CSF, IL-4, and IL-13 as described previously.^58^ Flow cytometry analysis of these cells did not reveal any changes in the expression of the M2 markers CD163, CD206, and CD209, in presence of IFN-*λ*4 (Fig. 3C, left). However, reduced *IL-10*, *RELM-β* (resistin-like beta), *ICAM*, and *VCAM* expression was observed (Fig. 3C, right). Both ICAM and VCAM adhesion molecules were included in the analysis as IFN-*λ*4-treated cells showed an increased tendency to clump in culture (Supporting Information Fig. S1c). These results suggest that IFN-*λ*4 can induce a subdued M1 phenotype in macrophage-like cells differentiated under M1 conditions, whereas the M2 phenotype is not significantly affected.

### 3.3 IFN-*λ*4 induces peripheral blood monocyte differentiation into a distinct phenotype of macrophages, whereas DCs show variable effects on Th cell cytokine responses

**Figure 4:**
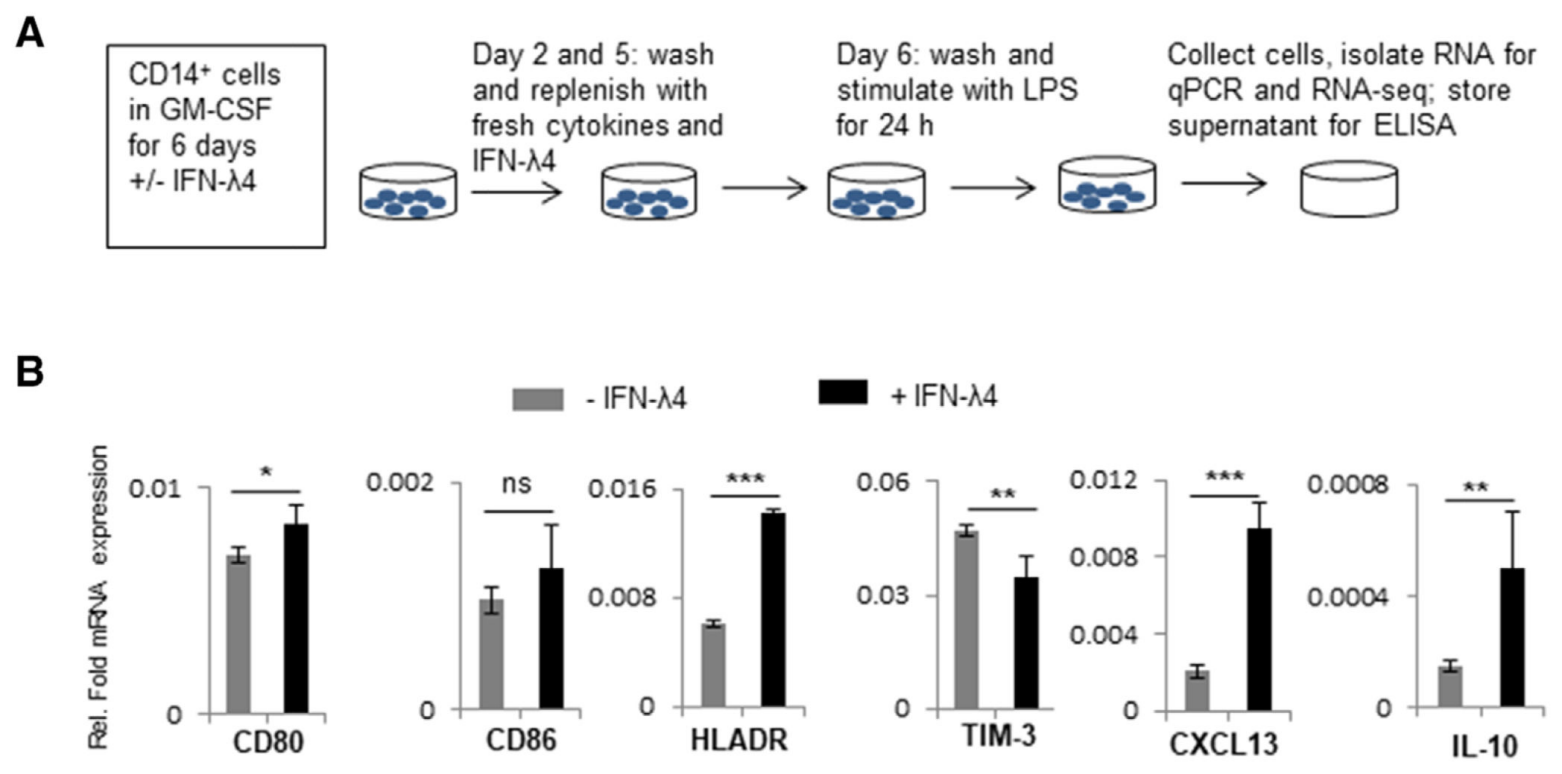
IFN-*λ*4 influences M1-monocyte-derived macrophages (MDM) differentiation. (**A**) Schematic representation of the experimental design of M1-MDM differentiation. Monocytes were obtained from a single donor. (**B**) The bar graph shows mRNA expression analysis by quantitative polymerase chain reaction (qPCR). The data show the mean and _SD_ from six technical replicates derived from two biological replicate experiments (*n* = 3 × 2). *P < 0.05; **P < 0.01, ***P < 0.001

**Figure 5:**
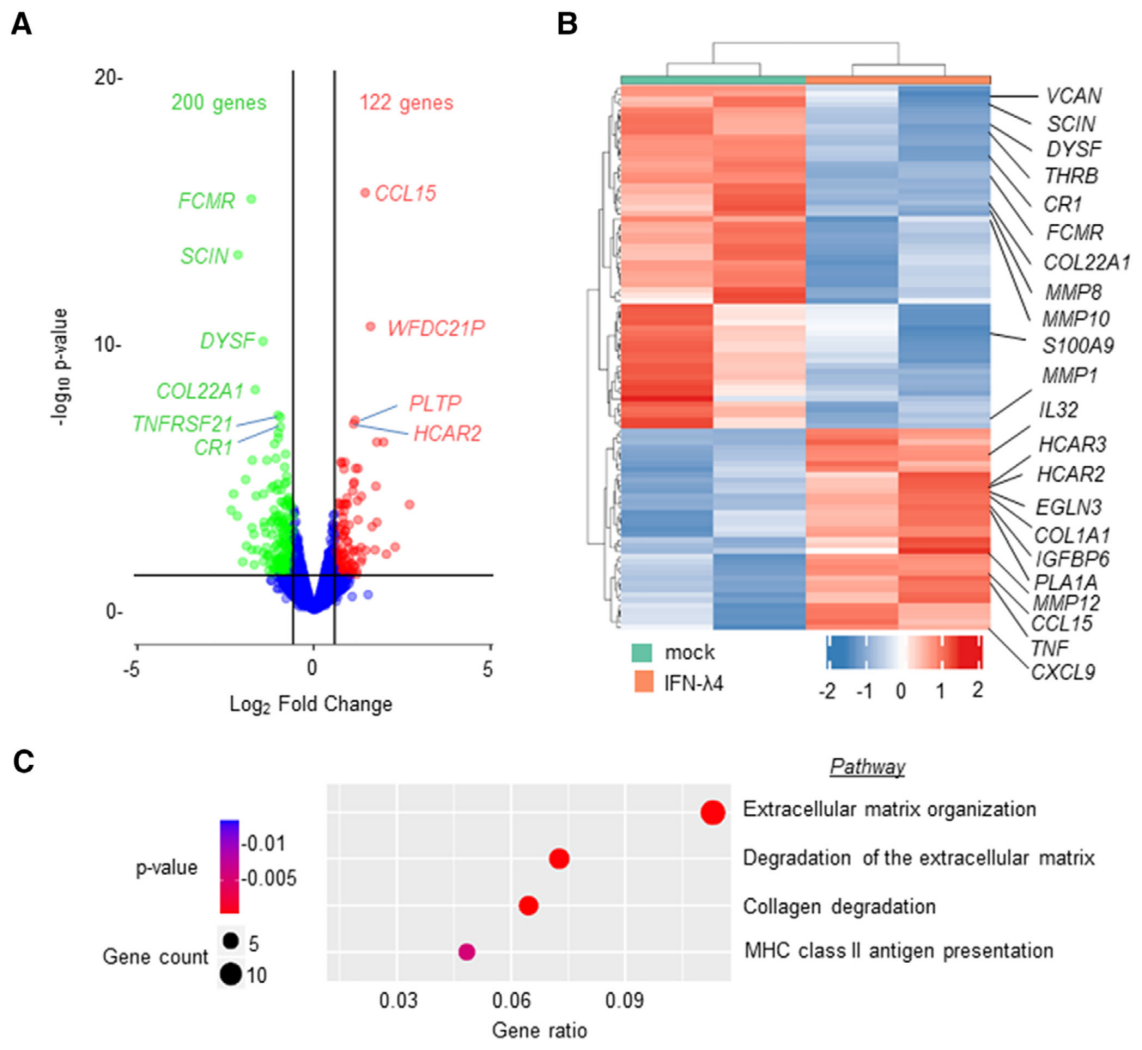
RNA-sequencing (RNA-seq) analysis shows that IFN-*λ*4 confers a modified M1 phenotype on monocyte-derived macrophages (MDMs). MDMs differentiated from CD14+ monocytes (as shown in Fig. 4A) from a single donor were subjected to paired-end RNA-seq analysis. Duplicate samples that had mock or IFN-*λ*4 treatment during differentiation with GM-CSF were used. (**A**) Volcano plot showing the differentially expressed genes (DEGs) in IFN-*λ*4-treated MDMs compared with untreated cells; a cut-off of 1.5-fold change and *P* = 0.05 were used. (**B**) Heatmap of the top 100 upand down-regulated DEGs are shown, with duplicate IFN-*λ*4-treated and mock-treated samples in different colors. Some of the important genes are shown on the right (also included in Table 1). (**C**) Reactome pathway enrichment analysis bubble plot of the DEGs (IFN-*λ*4 vs. mock) showing the top four most affected pathways

Because THP-1 is a cancer cell line and previous reports have shown that some inflammatory genes are conversely regulated in PMAdifferentiated THP-1 cells and MDMs,^59^ human peripheral blood CD14^+^ monocytes were used next to further examine the effect of IFN-*λ*4. CD14^+^ cells were obtained from a human donor (PromoCell) and were differentiated into M1-MDMs or MoDCs using GM-CSF alone or GM-CSF+IL-4, respectively, in the presence or absence of IFN-*λ* 4 according to established protocols.^10,44^ M1-MDMs were activated with LPS and subjected to mRNA expression analysis (Fig. 4). Surprisingly, several of the genes that were significantly differently expressed at the mRNA level in THP-1-derived macrophage-like cells treated with IFN-*λ*4 (Fig. 3B and C) showed a converse expression pattern in M1-MDMs (Fig. 4B). In particular, *CD80*, *CD86*, *HLA-DR*, and *CXCL-13* showed increased expression, whereas *TIM-3* was reduced, suggesting an enhanced M1 proinflammatory phenotype. Similar to the mixed phenotype observed in THP-1-derived macrophage-like cells, *IL-10*, an M2 marker, was significantly increased (Fig. 4B) in M1-MDMs differentiated in the presence of IFN-*λ*4. Thus, M1-MDMs also showed a mixed M1 phenotype (a phenotype that cannot be classified as a typical M1 or M2 phenotype). To gain further insights into the global gene expression changes in the M1-MDMs, RNA-seq analysis was performed in M1-MDMs (Fig. 5) differentiated in the presence or absence of IFN-*λ*4 and further activated for 24 h with LPS (Fig. 4A, for schematic). Overall, ∼700 genes were identified as being differentially expressed under the unadjusted *P*-value of 0.05, and the number reduced to ∼290 genes after false discovery rate correction. There were 322 genes that were either up- or down-regulated at a cutoff of 1.5-fold change at an unadjusted *P* = 0.05 (Fig. 5A), with the majority of these genes being down-regulated. Similar to the qPCR analysis, there were a number of up-regulated pro-inflammatory genes, including *TNFA* along with *CCL15*, *CXCL9*, and *IL32* (Table 1; Fig. 5B). Other M1 marker genes that were up-regulated included *EGLN3* (Egl-9 family hypoxia inducible factor 3 [PHD3]), and those that were downregulated included (scinderin) *SCIN* and others. Down-regulated M2 marker genes included *CD163*, versican (*VCAN*), and others (Table 1), whereas the M2 markers *CD1B* and *HCAR2* were up-regulated. Interestingly, several genes related to extracellular matrix (ECM) remodeling, such as *MMP1*, *8*, *10*, and *19*, were significantly down-regulated, and *COL1A1* and *COL22A1* were also affected. Genes considered important in diseases and homeostasis, such as *DYSF*, *IGFBP* (insulin like growth factor binding protein), *FCMR* (Fc fragment of IgM receptor), WAP four-disulfide core domain 21 (*WFDC21*), *PLTP* (phospholipid transfer protein), among others, were also significantly altered. Pathway analysis showed that the top three most affected pathways involving the DEGs included ECM remodeling processes (Fig. 5C), which suggested that IFN-*λ*4 could be important in fibrotic diseases where a genetic association with the *IFNL* locus has been recently established.^16,19–22^

Next, the effect of IFN-*λ*4 on DCs was tested. First, we confirmed that IFN-*λ*4 was able to influence THP-1-derived DCs (Supporting Information Fig. S2). THP-1 cells differentiated into DCs with GMCSF and IL-4^60^ were capable of responding to IFN-*λ*4 (Supporting Information Fig. S2a). We checked for expression of IFN-LR1 (IL28RA) expression using flow cytometry and then confirmed that IFN-*λ*4 was able to induce ISG expression in THP-1-derived DCs. We also subjected the DCs to cytokine analysis after activation with LPS, but saw only a marginal increase in TNF-*α*, but not IL-6 expression (Supporting Information Fig. S2b). We next examined the expression of HLA-DR and costimulatory molecule markers using flow cytometry (Supporting Information Fig. S2c). Unlike PMA-treated macrophage-like cells, DCs differentiated from THP-1 cells in the presence of IFN-*λ*4 showed a marked decrease in CD80, CD86, and HLA-DR marker expression. IFN-*λ*4 treatment also decreased overall CD209 marker expression as we observed the presence of two distinct populations of cells, one completely lacking CD209 expression. Expression differences at the mRNA level were only significant for *HLA-DR* (Supporting Information Fig. S2d).

**Figure 6:**
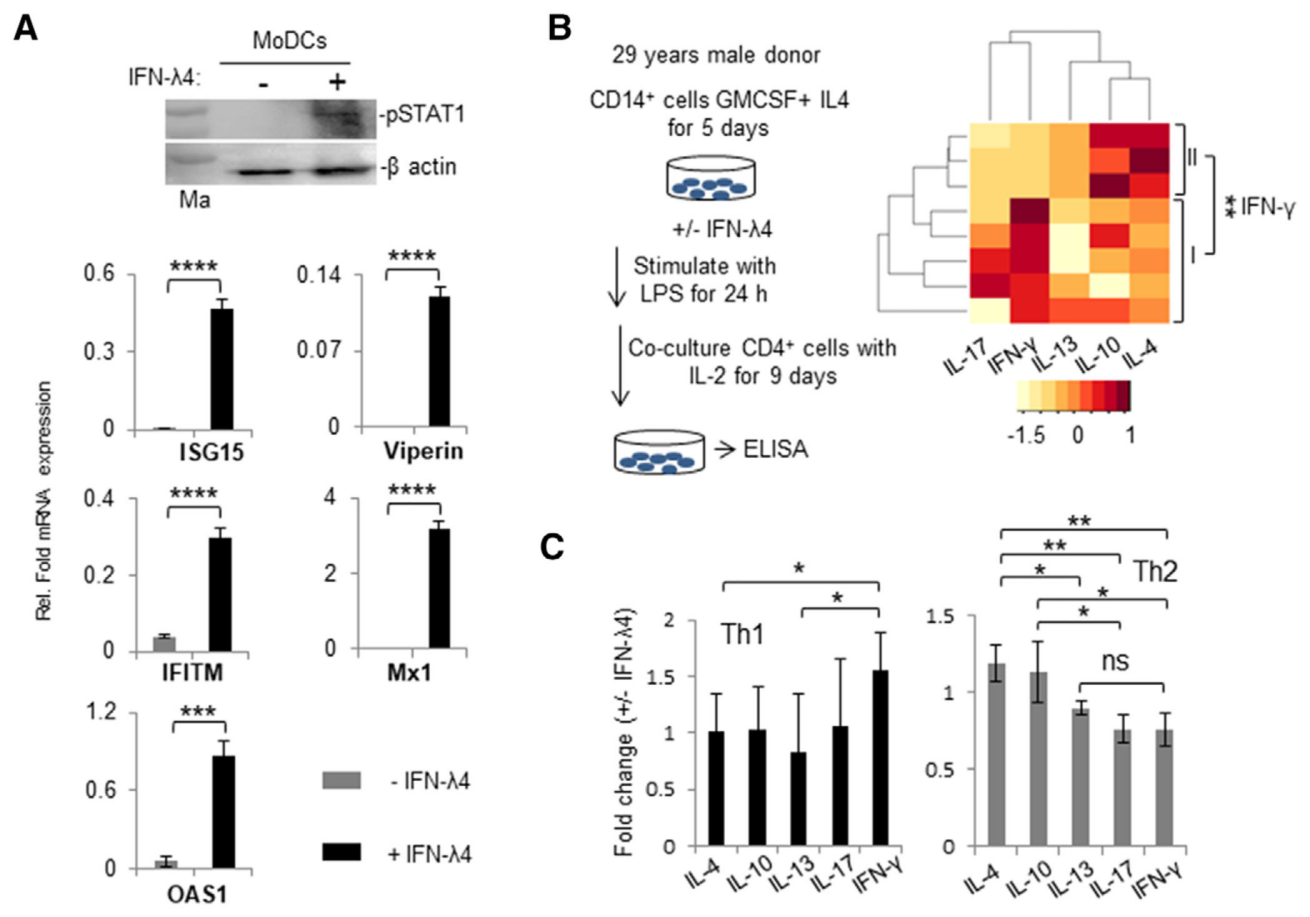
Monocyte-derived dendritic cells (MoDCs) respond to IFN-*λ*4 and when differentiated in its presence can alter cytokine expression from allogeneic T helper (Th) cells. (**A**) MoDCs respond to IFN-*λ*4. (top) CD14^+^ cells from a single healthy donor were isolated by positive selection as described in Section 2 (“Materials and Methods”) and differentiated into MoDCs in the presence of GM-CSF and IL-4 for 7 d. Afterward, recom-*β*-actin was used as a control. (Bottom) Quantitative polymerase chain reaction (qPCR) showing stimulation of IFN-stimulated genes by IFN-*λ*4 in MoDCs. MoDCs were generated as above for 7 d and recombinant IFN-*λ*4 (6 *μ*g/ml) was added or not for 24 h, and the total RNA isolated from the cells was used for qPCR analysis. The data show the mean of technical triplicates from one experiment with error bars depicting _SD_. ***P < 0.001; ****P < 0.0001. (**B**) The left portion shows a schematic representation of MoDC differentiation. The right portion shows the heatmap of fold change in secretion of different cytokines when IFN-*λ*4 (±) MoDCs were cocultured with CD4^+^ Th cells derived from eight unrelated allogenic donors. The color key at the bottom shows row *Z*-score. The donors could be separated in two clusters (I and II) based on fold changes in IFN-*λ* secretion. (C) The two groups of donors (I and II identified from **B**) were compared within themselves for fold changes involving different cytokines. IFN-*λ* represents a Th1 cytokine, and IL-4 and IL-13 represent Th2 cytokines. The data show mean average fold change from eight coculture experiments (with MoDCs from a single donor and Th cells from eight donors) with error bars depicting _SD_. For (**A**), (**B**), and (**C**): *P < 0.05, **P < 0.01, ***P < 0.001

Next, MoDCs were obtained in the presence or absence of IFN-*λ* 4 using established protocols.^10^ We first confirmed that MoDCs responded to IFN-*λ*4 by examining the phosphorylation of STAT1 and promotion of ISGs expression in treated cells (Fig. 6A). In a separate experiment using CD14^+^ cells from a single human donor (PromoCell), MoDCs were generated and used in a coculture to stimulate CD4^+^ Th cells that were isolated from eight unrelated donors. A suite of cytokines released in the coculture media were evaluated after stimulation of the CD4^+^ Th cells with PMA and ionomycin (Fig. 6B).^10^ MoDCs differentiated in presence of IFN-*λ*4 did affect the cytokine release pattern from the CD4^+^ Th cells; however, there was no significant effect on any of the cytokines released when all eight donor cells were considered together (Supporting Information Fig. S3a). A clustering algorithm identified two groups of Th cell donors (identified as I and II in Fig. 6B heatmap) based on their fold change in IFN-*γ* expression (Fig. 6B and Supporting Information Fig. S3b). Further analysis revealed that the first group showed a typical Th1 response and the second had a typical Th2 response pattern (Fig. 6C).

### 3.4 Recombinant IFN-*λ*4 induces lower expression of ISGs in immune cells compared with IFN-*λ*3

**Figure 7:**
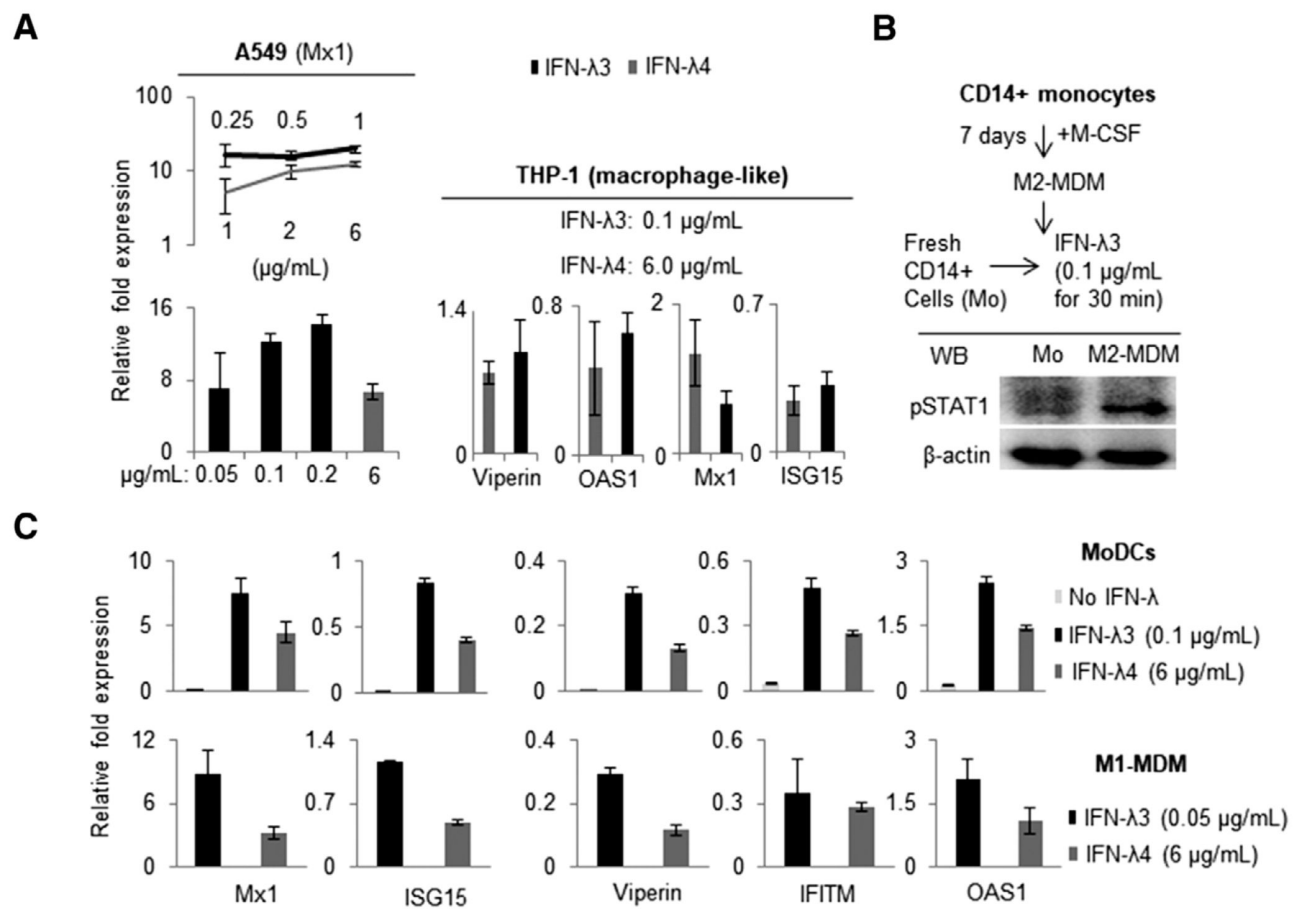
Recombinant IFN-*λ*3 shows superior specific activity compared to recombinant IFN-*λ*4 in IFN-stimulated genes (ISGs) stimulation in various cell types. (**A**) A range of concentrations were tested for the two cytokines in A549 (left; procedure followed same as in Fig. 2C) and PMA-differentiated THP-1 cells (right; THP-1 cells were treated with PMA for 48 h and then treated with IFN-*λ*3 or IFN-*λ*4 for 24 h) and quantitative monocyte-derived macrophage (MDM) cells generated from human PBMC-derived CD14^+^ cells (obtained by negative selection) as described in polymerase chain reaction (qPCR) was carried out to measure the ISG expression. (b) Western blot showing the expression of pSTAT1 in M2 Section 2 (“Materials and Methods”). (**C**) ISG stimulation activity of IFN-*λ*3 and IFN-*λ*4 was tested at the given concentrations in in vitro generated monocyte-derived dendritic cells (MoDCs) and M1-MDMs from a single donor as described in Section 2 (CD14^+^ monocytes were isolated from PBMCs of a healthy volunteer by positive selection for experiments shown in (**C**) as described in Section 2. For **A** and **C**: The data show mean from technical triplicates from one experiment with error bars depicting SD

Because the study aimed to address the contrasting effect of IFN-*λ*4 vs. IFN-*λ*3 on immune cells, it was necessary to ascertain a comparable concentration of the two cytokines that could be used (Fig. 7). Recombinant IFN-*λ*3 was also commercially obtained and used first in A549 cells along with IFN-*λ*4 to measure ISG stimulation activity (Fig. 7A). When tested in a range of concentrations, marked differences in the specific activity of recombinant IFN-*λ*3 and IFN-*λ*4 was observed, with IFN-*λ*3 showing very high ISG stimulation activity even at much lower concentrations compared with IFN-*λ*4 (Fig. 7A, left). Afterward, a 60-fold lower concentration of IFN-*λ*3 compared with IFN-*λ*4 was used on PMA-differentiated THP-1 cells (Fig. 7A, right) and the expression of several ISGs was measured by qPCR. At these selective concentrations, IFN-*λ*4 reached an activity similar to IFN-*λ*3 regarding some ISGs. In vitro generated M2-MDMs incubated with 0.1 *μ*g/ml IFN-*λ*3 showed strong STAT1 phosphorylation (Fig. 7B), whereas a similar treatment to freshly isolated monocytes did not induce STAT1 activation. These results are in agreement with previous reports^12,33,37,38,41,42,44^ describing that PBMC-derived primary human CD14^+^ monocytes are incapable of responding to IFN-*λ*s. Moreover, a concentration of 0.1 *μ*g/ml, and even 0.05 *μ*g/ml, of IFN-*λ* 3 was still high when compared to IFN-*λ*4 at 6 *μ*g/ml in MoDCs and M1-MDMs (Fig. 7C). A recent study used a concentration of 0.1 *μ*g/ml of IFN-*λ*3 to examine the effect on macrophage differentiation.^12^ Nevertheless, based on the results presented here, 0.05 *μ*g/ml of IFN-*λ* 3 was selected to be used in the remaining experiments to compare its effect with IFN-*λ*4 at 6 *μ*g/ml, even though the ISG stimulation activity of the two cytokines was still not comparable at these concentrations.

### 3.5 M1 macrophages differentiated in the presence of IFN-*λ*3 or IFN-*λ*4 show differences in IL-10 expression

**Figure 8:**
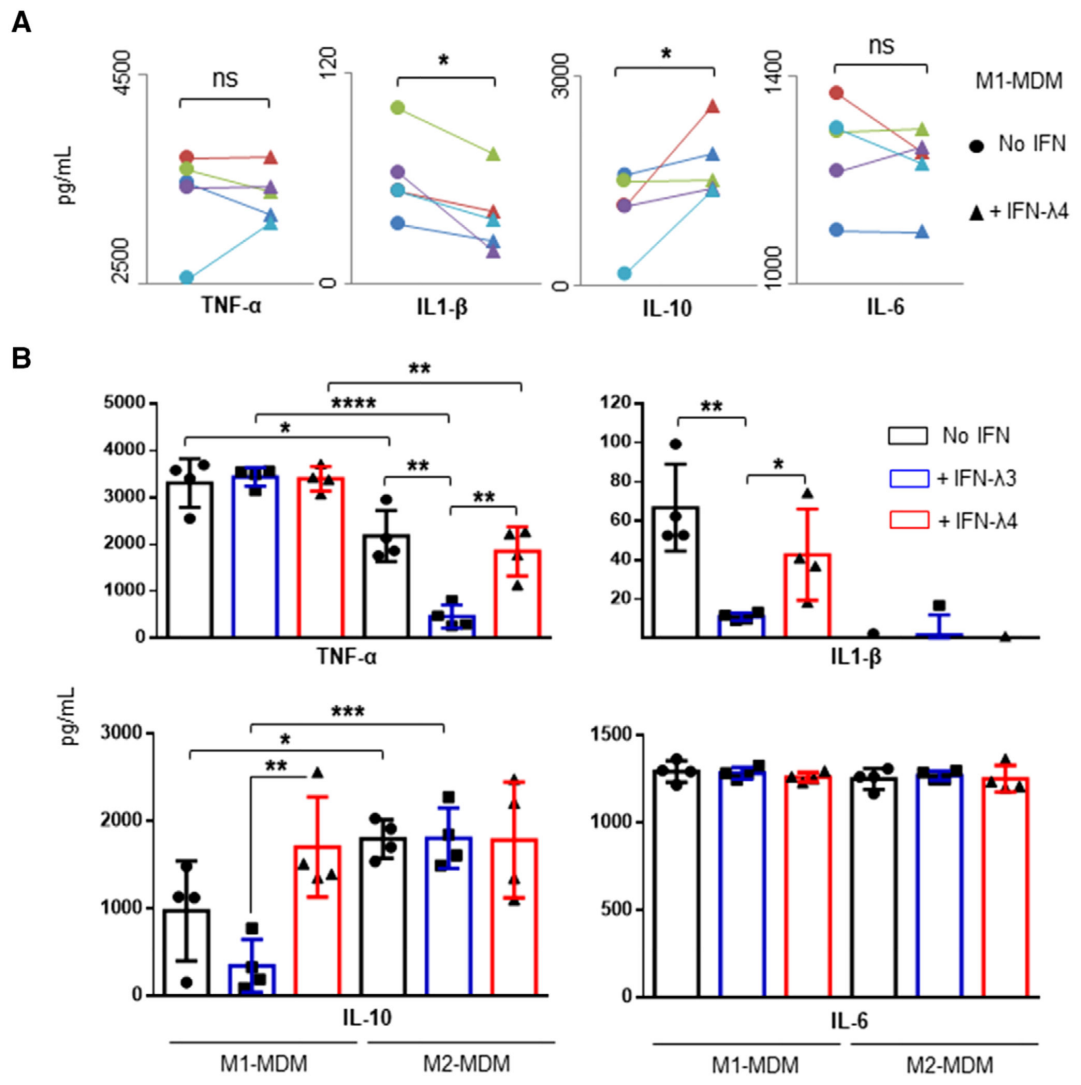
Monocyte-derived macrophages (MDMs) differentiated in the presence of IFN-*λ*3 or IFN-*λ*4 show altered cytokine secretion. (**A**) Activated M1-MDMs differentiated in the presence of IFN-*λ*4 show lower IL-1*β* and higher IL-10 secretion. CD14^+^ cells obtained from five independent donors were differentiated into M1-MDMs and stimulated with LPS (as per scheme in Fig. 4A), and cytokine secretion was measured MDMs derived from each of the five donors (in different colors) differentiated without and with IFN-*λ*4 respectively, joined by a trend line. A 1by ELISA. The data show mean values from five independent donors with error bars representing _SD_. Filled circles and filled triangles represent tailed *t*-test for two dependent means was carried out to calculate statistical significance. *P < 0.05; ns, not significant. (**B**) Cytokine profiles of activated M1and M2-MDMs differentiated in the presence of IFN-*λ*3 or IFN-*λ*4. The data show mean values from four independent donors with error bars representing _SD_. CD14^+^ cells from each donor were split into three aliquots and differentiated into M1or M2-MDMs in the absence or presence of IFN-*λ*3 or IFN-*λ*4; after activation with LPS, cytokines were collected from supernatants and measured by ELISA. A 2-tailed *t*-test for two independent means was used to calculate statistical significance; only significant comparisons are shown; *P < 0.05, **P < 0.01, ***P < 0.001, ****P < 0.0001

Using a single CD14^+^ cell donor, IFN-*λ*4 was found to affect the differentiation process of M1-MDMs (Figs. 4 and 5) as well as the expression of several genes at the mRNA level. Next, the expressions of cytokines at the protein level were also evaluated in differentiating macrophages upon IFN-*λ*4 treatment. CD14^+^ cells from four more donors (PromoCell) were differentiated into M1-MDMs in the presence or absence of IFN-*λ*4 and further stimulated with LPS, and the expression of some important cytokines were assessed by ELISA (Fig. 8A). Although the secretion of TNF-*α* and IL-6 was not affected by IFN-*λ*4 treatment, M1-MDMs differentiated in the presence of IFN-*λ*4 showed significantly lower IL-1*β* and higher IL-10 expression (Fig. 8A), suggesting that IFN-*λ*4 has an overall anti-inflammatory effect on differentiating M1 macrophages.

To address the contrasting effect of IFN-*λ*3 and IFN-*λ*4 on differentiating macrophages, the CD14^+^ cells obtained from the four donors (PromoCell) were separately differentiated into M1 and M2-MDMs in the presence or absence of 0.05 *μ*g/ml IFN-*λ*3 or 6 *μ*g/ml IFN-*λ*4, and then stimulated with LPS. A new analysis of the cytokine secretion profiles of the stimulated M1 and M2-MDMs (Fig. 8B) revealed that, even though IFN-*λ*3 was used at a 120-fold lower concentration than IFN-*λ*4, its effect on the secretion of cytokines was more pronounced. Notably, MDMs differentiated in the presence of IFN-*λ* 3 showed a decrease in the secretion of TNF-*α* under M2 conditions and IL-1*β* under M1 conditions. MDMs differentiated in the presence of IFN-*λ*4 also showed similar trends, but were less pronounced. The most interesting result was observed in IL-10 secretion from M1-MDMs. Although MDMs differentiated in the presence of IFN-*λ*3 or IFN-*λ*4 altered the secretion of TNF-*α* and IL-1*β* from M2-MDMs and M1-MDMs, respectively, in similar directions, their effect on IL-10 secretion from M1-MDMs was the opposite (Fig. 8B). M1-MDMs differentiated in the presence of IFN-*λ*3 showed reduced secretion of IL-10, whereas M1-MDMs differentiated in the presence of IFN-*λ*4 showed increased IL-10 secretion. No effect from either IFN-*λ*3 or IFN-*λ*4 was seen on the secretion of IL-6 in M1 or M2-MDMs.

### 3.6 Comparison of cytokine secretion profiles from allogenic Th cell cocultures with MoDCs differentiated in the presence of IFN-*λ*3 or IFN-*λ*4

**Figure 9:**
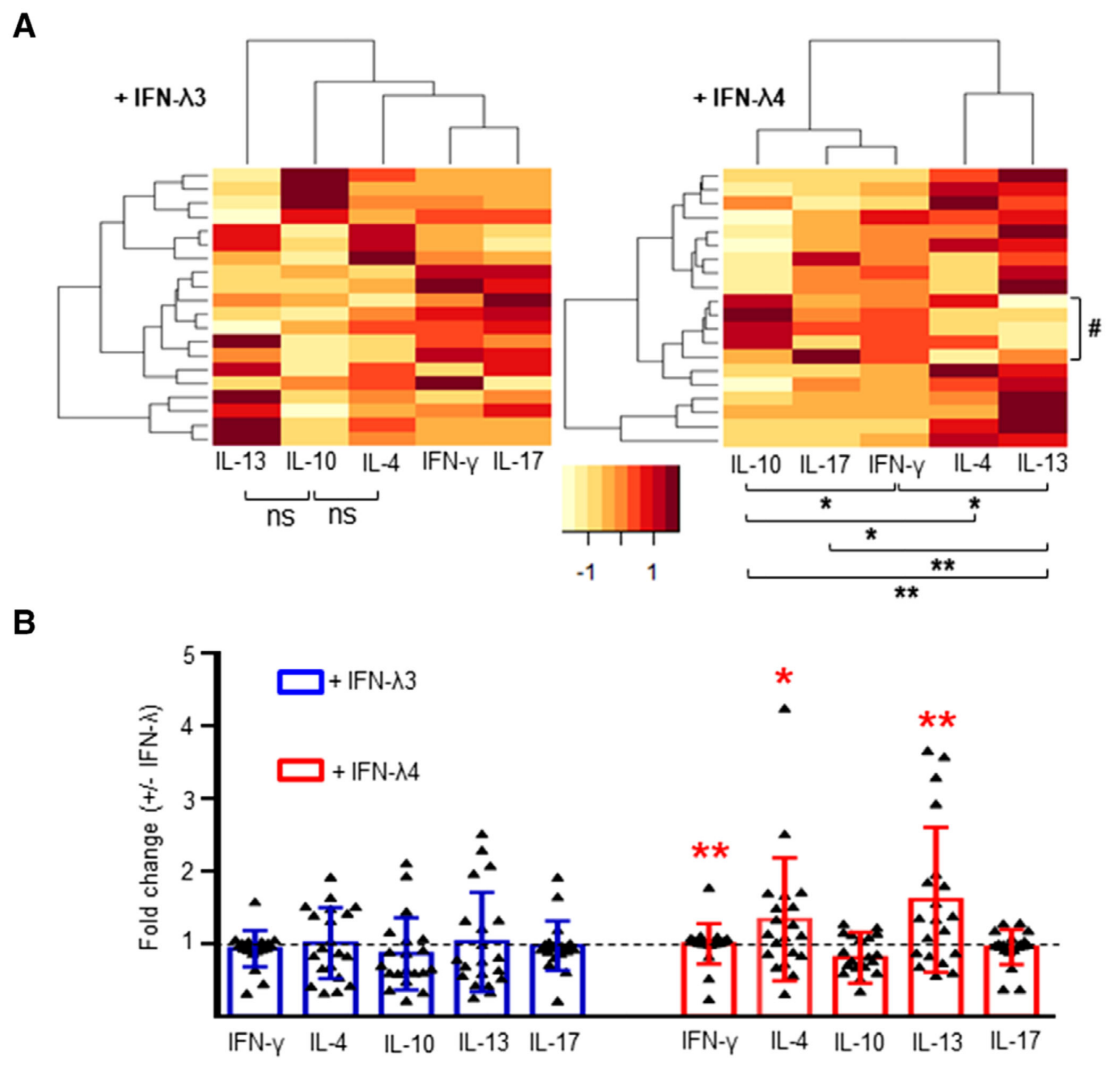
Comparison of fold changes in cytokine secretion in cocultures of CD4^+^ cells and monocyte-derived dendritic cells (MoDCs) differentiated in the presence of IFN-*λ*3 or IFN-*λ*4. MoDCs were differentiated from CD14^+^ cells obtained from four donors in the absence or presence of IFN-*λ*3 or IFN-*λ*4; coculture experiments were set up with CD4^+^ cells from 20 allogenic donors and cytokines were collected from supernatants ated in the presence of IFN-*λ*3 (left) or IFN-*λ*4 (right) and allogenic CD4^+^ cells. The color key at the bottom shows row *Z*-score. The comparisons and measured by ELISA. (**A**) Heatmap showing the fold changes of five cytokines collected from coculture supernatants involving MoDCs generComparison of cytokine profiles between 20 allogenic CD4^+^ donors in coculture experiments involving MoDCs derived in presence of IFN-*λ*3 or shown below the heatmap are between any two cytokines by paired *t*-tests. Only the comparisons that were statistically significant are shown. (**B**) IFN-*λ*4. The comparison is between IFN-*λ*3 and IFN-*λ*4 for a given cytokine. The bars depict mean values and error bars depict _SD_. A 2-tailed *t*-test significant differences in their means for IFN-*λ*3; for IFN-*λ*4, however, a 1-tailed *t*-test showed significance in some new comparisons: IFN-*γ* vs. IL-4 for dependent means was used for all statistical comparisons in (**A**) and (**B**); a 1-tailed *t*-test did not show any new groups of cytokines that had (*P* = 0.03) and IL-4 vs. IL-17 (*P* = 0.03). For (**A**) and (**B**) *P < 0.05, **P < 0.01; ns, not significant

The cytokine profiles of cocultures of allogeneic CD4^+^ cells and MoDCs differentiated in the absence or presence of IFN-*λ*3 or IFN-*λ*4 were compared (Fig. 9). MoDCs obtained from four CD14^+^ cell donors (PromoCell) were cocultured with CD4^+^ cells obtained from five different donors, resulting in a total of 20 CD4^+^ cell donors (Supporting Information Fig. S4, top scheme). Among them, 10 donors belonged to a younger age group (median age: 28 yr) and a higher socioeconomic status, and 10 donors belonged to an older age group (median age: 43 yr) and a lower socioeconomic status. Initial comparisons were performed without accounting for the differences between the two groups. In this approach, it was revealed that MoDCs raised in the presence of IFN-*λ*3 did not have an overall significant effect on the CD4^+^ cells from the 20 individuals, such that Th1 or Th2 skewing took place (Fig. 9A). MoDCs raised in the presence of IFN-*λ*4, however, showed significant differences in the levels of cytokines secreted from the CD4^+^ cell coculture experiments derived from the 20 donors (Fig. 9A). In contrast to the previous results shown in Figure 6B and C, it was not possible to separate the CD4^+^ cell donors into clusters based on either IFN-*λ* or other cytokine secretion differences. Nevertheless, five individuals showed a Th1 response (IFN-*λ* vs. IL-13 [*P* = 0.01]; marked by # in Fig. 9A in the heatmap involving IFN-*λ*4), but there were several other significant differences in the fold changes involving the five cytokines, in particular among IL-10 and IL-13 (Fig. 9A). There was significant up-regulation of IL-13 and, to a lesser extent, IL-4, compared with IFN-*λ*, IL-17, and IL-10, suggesting an overall Th2 phenotype (Fig. 9A and B). In contrast, MoDCs raised in the presence of IFN-*λ*3 induced no significant overall fold changes in any of the five cytokines secreted by the CD4^+^ cells interacting with them (Fig. 9A and B). CD4^+^ cell ability to secrete IFN-*λ* , IL-4, and IL-13 significantly increased when interacting with MoDCs obtained in the presence of IFN-*λ*4 compared with those cultured in the presence of IFN-*λ*3 (Fig. 9B).

By analyzing the data based on the age groups of the CD4^+^ cells donors it was possible to observe that MoDCs differentiated in the presence of IFN-*λ*3, unlike in the combined analysis, induced significant differences in the expression of IL-4 and IL-13 by CD4^+^ cells from younger vs. older donors (*n* = 10 each), whereas IFN-*λ*4 seemed to affect only IL-17 secretion (Supporting Information Fig. S4a). Furthermore, MoDCs differentiated in the presence of IFN-*λ*3 or IFN-*λ* 4 showed significant differences in fold changes in the secretion of IFN-*γ*, IL-4, and IL-13 in cocultures involving CD4^+^ cells obtained from younger but not older donors (Supporting Information Fig. S4b).

The most valid comparisons for differences in the effect of IFN-*λ* 3 and IFN-*λ*4 on MoDC differentiation can be made only if taken into consideration both the CD4^+^ and the CD14^+^ cell donor characteristics. Therefore, the effect on cytokine secretion was compared in cocultures involving MoDCs derived from the four CD14^+^ donors separately (with *n* = 5 CD4^+^ cell donors each) (Supporting Information Fig. S4c and d). IFN-*λ*3 and IFN-*λ*4 (through MoDCs) did not had a significantly different effect on cytokine secretion from cocultures that had as CD14^+^ donors the C or D individuals (Supporting Information Fig. S4d); however, the CD14^+^ donors A or B showed significant differences on cytokine secretion in the cocultures (Supporting Information Fig. S4c). Significant differences in fold change in IFN-*λ* and IL13 secretion were observed between IFN-*λ*3 and IFN-*λ*4 in cocultures involving CD14^+^ cells from donor A. Moreover, if the CD14^+^ cells were from donor B, then significant differences were observed in IFN-*λ*, IL-4, and IL-13 secretion (Supporting Information Fig. S4c). In both cases, IFN-*λ*4 was associated with a higher level of fold change of the respective cytokines compared with IFN-*λ*3. However, a closer examination of the data suggests that IFN-*λ*3 contributed more to the association by causing an overall significant down-regulation of cytokines, especially with results involving donor B, whereas IFN-*λ*4 was also driving the association actively by up-regulating IL-4 and IL-13 (Supporting Information Fig. S5). This analysis suggests that the CD14^+^ cell donor background may be an important determinant in the differential effects of IFN-*λ*3 and IFN-*λ*4 on cytokine secretion pattern. Nonetheless, it is possible that the two different age groups of the CD4^+^ cell donors could be acting as potential confounders in the observed results.

In summary, from the results presented in Figure 9 and Supporting Information Figures S4 and S5, we conclude that IFN-*λ*3 and IFN-*λ*4 can affect the differentiation of MoDCs such that their subsequent interaction with allogenic Th cells may be significantly affected, as seen by an effect on cytokine secretion by Th cells in cocultures. Although age and gender of the donor (CD14^+^ cell donor A was a female, whereas B, C, and D were males) may modify the impact of IFN-*λ*3 such that no overall effect on Th cells is seen, under similar circumstances, IFN-*λ*4 shows a Th2-biased phenotype (Fig. 9).

## 4 Discussion

IFN-*λ*4 has been studied as an antiviral cytokine^1–7^; however, the genetic association of the dinucleotide polymorphism rs368234815, which is responsible for IFN-*λ*4 expression, extends beyond viral infections, including several inflammatory disorders,^15–22^ and even parasitic infections^27^ and cancer.^28–30^ Two SNPs are known to potentially regulate the expression of IFN-*λ*3^46,47^ and it is not clear in many of the genetic associations which among IFN-*λ*3 and IFN-*λ*4 could be the causal factor.^8,13^ . By using statistical methods, some studies have shown that it is IFN-*λ*4 and not IFN-*λ*3 that could be the causal factor behind the genetic association of the IFN-*λ* locus with HCV and other infections.^27,61^ Other studies have used the phenotypic variation associated with a nonsynonymous variant rs117648444 within *IFNL4* in combination with functional data to show that IFN-*λ*3 but not IFN-*λ*4 is the causal factor behind HCVassociated liver fibrosis.^20^ The genetic association of the IFN-*λ* locus variants with human health^62^ and disease conditions^63^ is increasingly being recognized.^14^ Moreover, the IFN-*λ* genes are now recognized as potential pivotal players in the development of the immune system. Therefore, it is important to fully decipher the functions of this new class of IFNs. IFN-*λ*1 is the most studied of all type III IFNs for its nonantiviral functions.^10,11,34,38–40^ Although some studies, mainly from the Gallagher group, have characterized IFN-*λ*1 as a modulator of Th cells toward Th1 phenotype,^10,11,39,40^ others have shown that it drives them to a Treg phenotype.^38^ A very recent report suggested that IFN-*λ*3 could also have features of a Th1-like IFN, even though its evidences were based on IFN-*λ*3-treated MDMs.^12^ In this report, Read et al. described the immunomodulatory properties of IFN-*λ*3 on in vitro differentiated M1- and M2-MDMs, finding that IFN-*λ*3 could induce most of M1 markers but not M2 markers. Interestingly, they followed the same pretreatment strategy used herein (Fig. 2A) to show that several Th1-related chemokines, such as CXCL3, 5, 10, and others, were induced more strongly in M1-MDMs than in M2-MDMs after differentiating in the presence of IFN-*λ*3. They further showed that MDMs differentiated in the presence of IFN-*λ*3 had increased phagocytic and cytotoxic capacity, and to promote lymphocyte migration and NK cell degranulation. Similar to our study, they performed global gene expression profiling of MDMs that were differentiated in the presence of IFN-*λ*3. However, there was a notable difference in the strategy followed in their study and ours: they characterized the MDMs differentiated in the presence of IFN-*λ*3 without activating them, potentially to avoid losing sensitivity to IFN-*λ*3 treatment due to skewing of the phenotype, whereas we have carried out all our experiments with LPS-stimulated MDMs (Figs. 4, 5 and 8). Not surprisingly, they found a number of ISGs that were upregulated in their IFN-*λ*3-treated MDMs, whereas we did not find any IFN-related genes except *NRIR* (negative regulator of IFN response [nonprotein coding], fold change: 3.09, false discovery rate corrected: *P* = 0.08), out of the 322 genes whose expression was significantly (unadjusted *P* < 0.05) changed by a factor of more than 1.5-fold by IFN-*λ*4. Our RNA-seq data obtained from LPS-stimulated IFN-*λ*4-differentiated M1-MDMs showed many genes related to inflammation and ECM remodeling pathways and several disease-related genes that were affected (Table 1 and Fig. 5; Ex. *DYSF*,^64^ *IGFBP*,^65^ *FCMR*,^66^ *WFDC21*,^67^ and *PLTP* ^68^). In the study by Read et al., MDMs differentiated in the presence of IFN-*λ*3 for 7 d were directly subjected to mRNA expression analysis, whereas in our study, after differentiating the MDMs in the presence of IFN-*λ*4 for 6 d, they were activated with LPS in the absence of growth factors and IFN-*λ*4 for 24 h and only then subjected to transcriptomics. Thus, due to the different strategies adopted, our results on gene expression in IFN-*λ*4-differentiated MDMs reflect a more stably altered transcriptomic state of the cells than the results of Read et al., which may reflect a more transient antiviral state induced by the presence of IFN-*λ*3 during the process of differentiation.

This study was conducted with the primary objective of characterizing any modulatory effect that IFN-*λ*4 could have on immune cells. Moreover, it also aimed to perform a comparative analysis between IFN-*λ*3 and IFN-*λ*4. To this end, firstly a strategy was established to address the question of whether IFN-*λ*4 affects the phenotypes of macrophages and DCs. THP-1 cells were used in the initial experiments, demonstrating that the pretreatment strategy (Fig. 2A) was ideal to study the modulatory effect of IFN-*λ*4 on macrophage-like cells and DCs. This also suggested that the effect of IFN-*λ*4 on the differentiated macrophage-like cells was not transient and likely to have occurred at an epigenetic level because the final stimulation phase with LPS for 24 h did not had IFN-*λ*4 in the cell culture media (Fig. 2A). THP1 cells themselves showed to not respond to IFN-*λ*4, perhaps due to a lack of IFN-LR1 and STAT1 expression, but PMA treatment made them responsive (Fig. 1C and D). An initial analysis of cytokine secretion suggested that IFN-*λ*4-treated macrophage-like cells had a proinflammatory phenotype (Fig. 2B and D), but later analysis on M1 and M2 specific marker expression showed that IFN-*λ*4 rendered them a subdued M1 phenotype, which we refer to as a mixed phenotype, because both M1 and M2 phenotypes were observed (Fig. 3A–C). Using IFN-*λ* 4-treated M1-MDMs derived from primary cells of a single donor, we again observed a mixed phenotype wherein several proinflammatory markers, such as *CD80*, *CD86*, *HLADR*, and *CXCL13*, but also *IL-10*, an anti-inflammatory cytokine marker, were up-regulated at the mRNA level (Fig. 4B). Our RNA-seq results agreed with a mixed phenotype wherein several proinflammatory and anti-inflammatory genes were affected in both directions (Table 1).

In a later analysis, which included a greater number of samples (*n* = 5), it was possible to observe the anti-inflammatory nature of M1-MDMs that were differentiated in the presence of IFN-*λ*4 (Fig. 8A). Importantly, IFN-*λ*4 showed an up-regulation of IL-10, an important protective cytokine in inflammatory disease conditions, such as liver fibrosis (Fig. 8A).^69,70^ This result potentially explains the protective effect seen in the IFN-*λ*4-generating ΔG allele carriers at rs368234815 in the case of several inflammatory diseases,^13,16,19^ including HCV-mediated liver fibrosis.^20^ Our RNA-seq results endorse this by showing that the three most affected pathways in IFN-*λ*4-treated M1-MDMs are related to ECM remodeling (Fig. 5C). It is possible that the expression of an active IFN-*λ*4 offers a protective effect in inflammatory conditions by increasing the secretion of anti-inflammatory cytokines such as IL-10 and by down-regulating matrix metalloproteinases, thereby circumventing a highly inflammatory environment in injured tissue so that subsequent healing of the wound can occur with minimal remodeling.

Comparing the effect of IFN-*λ*4 with that of IFN-*λ*3 on immune cells prompted the need of a working concentration of the two IFNs that could provide a comparable setting. However, detailed analysis showed that the recombinant IFN-*λ*4 used had very low specific activity compared with IFN-*λ*3 (Fig. 7). This result contrasted with previous reports showing that, under more physiologic conditions, IFN-*λ*4 can have higher specific activity than IFN-*λ*3, despite the fact that IFN-*λ* 4 is not efficiently secreted out of the cells.^9^ We believe that issues related to suboptimal folding of the recombinant IFN-*λ*4 during its purification process may be the reason for its relatively poorer specific activity. A concentration of IFN-*λ*3 that was approximately 120fold lower than of IFN-*λ*4 was selected for further use after a range of experiments performed in different cells (Fig. 7). Even at such a relatively low concentration, the effect of IFN-*λ*3 was more pronounced on cytokine secretion activity from both M1 and M2-MDMs (Fig. 8B). Interestingly, and in contrast to the conclusions drawn by Read et al.,^12^ we found that IFN-*λ*3 has an inhibitory effect on the secretion of two proinflammatory and one anti-inflammatory cytokines in our experiments (TNF-*α* under M2, IL-1*β* and IL-10 under M1 MDM conditions; Fig. 8B). As mentioned earlier, in contrast to the strategy used herein, the MDMs in the study by Read et al. were assessed without stimulation. Whether this difference could be responsible for the discrepant results remains to be clarified. Although IFN-*λ*4 also affects TNF-*α* and IL-1*β* similarly to IFN-*λ*3 in MDMs, albeit to a lower extent possibly due to its low specific activity, its effect on IL-10 is notably in the opposite direction (Fig. 8B). This would again explain why ΔG allele carriers at rs368234815 show a protective phenotype for several, if not all, inflammatory disease conditions.^13^

Coculture experiments allowed comparing the relative effects of IFN-*λ*3 and IFN-*λ*4 on MoDCs (Fig. 9). Although there was a clear Th2 bias in cytokine secretion from CD4^+^ cells when MoDCs were differentiated in the presence of IFN-*λ*4, such effect was not observed when MoDCs were obtained in the presence of IFN-*λ*3 (Fig. 9A and B). Further analysis showed that an interaction between the CD4^+^ and CD14^+^ cell donor background (age and gender) prevented us from appreciating a significant effect of IFN-*λ*3 in the cytokine secretion pattern (Supporting Information Fig. S4). Interestingly, because the same conditions were used when MoDCs were differentiated in presence of either IFN-*λ*3 or IFN-*λ*4, such an interaction of the donor background did not prevent us from appreciating an effect of IFN-*λ*4, which seemed to be toward a Th2-biased phenotype (IL-4 vs. IFN-*γ*, *P* = 0.03; IL-13 vs. IFN-*γ*, *P* = 0.005; Fig. 9B). Genetic studies have shown that similar to its effect on HCV infections, the ΔG allele has a proviral effect in other respiratory viral infections and a similar risk phenotype in malaria infections.^25,26,27^ The results shown here ascribe an over- all Th2 phenotype to IFN-*λ*4, which may explain the above mentioned genetic association studies. We do note that our results from Figure 6B show an overall Th1 phenotype associated with IFN-*λ*4, whereas the results in Figure 9 show it to have a Th2 phenotype. However, the results from Figure 9 are more reliable because those from Figure 6B are based on a single CD14^+^ cell donor (MoDCs derived from them interacted with Th cells from eight donors), whereas those shown in Figure 9 are from four separate CD14^+^ cell donors (MoDCs derived from them interacted with Th cells from five donors each). Moreover, we observed a similar Th2 bias for IFN-*λ*4 when a combined analysis of results from both Figures 6B and 9 was performed, albeit with a lesser significance level (IFN-*γ* vs. IL-13, *P* = 0.04; IFN-*γ* vs. IL-4 not significant; 1-tailed *t*-test for dependent means). Another potential factor affecting our results that we have not addressed in this report could be the genetic background of the donors at the IFN-*λ*4-generating polymorphism rs368234815.

The influence of age and gender on the genetic association of the IFN-*λ* locus with several human phenotypes has been noted previously.^15,16,21,25^ Our limited set of experiments in this report cannot address the issue of the modifying effect of age and gender on IFN-*λ* 3 and IFN-*λ*4 immunomodulatory functions. Additional well-designed and well-powered cohort studies are still warranted to clarify this issue.

The present study also confirmed previous reports describing monocytes as incapable of IFN-*λ* signaling^37,38,41,42^ (Fig. 7B).Our results also provide some clarification on the issue of IFN-*λ* signaling in DCs (Figs. 6A and 7), as previous reports have provided contradictory results.^38,42^

IFN-*λ*4 is not known to be widely expressed in tissues other than in HCV-infected livers. The drawback of this work is that it does not address the natural expression of IFN-*λ*4 in human cells and tissues so that the described effect could have physiologic significance. However, recent evidence has shown that IFN-*λ*4 can be expressed from ΔG alleles in nonhepatic cells,^29^ and further research is needed in this area.

In summary, this study provides the first evidence on IFN-*λ*4 immunomodulatory functions beyond its role as an antiviral cytokine. Future research is required to better understand this phenomenon in order to appreciate IFN-*λ* biology in humans from an immunogenetics perspective in both health and disease.

## Supplementary Material

Supporting Information Table 1

Supporting Information fig 1

Supporting Information fig 2

Supporting Information fig 3

Supporting Information fig 4

Supporting Information fig 5

## Figures and Tables

**Table 1:** Important genes identified by RNA-sequencing that were found to be differentially regulated after IFN-*λ*4 treatment to differentiating monocyte-derived macrophages (MDMs)

Sl. no.	Gene symbol	Gene name	Fold change	P-value	FDR
1	*TBC1D3K*	TBC1 domain family; member 3K	257.8223	5.60E-14	1.11E-10
2	*EGLN3*	Egl-9 family hypoxia-inducible factor 3	3.896497	4.30E-07	0.000295
3	*ADGRG2*	Adhesion G protein-coupled receptor G2	3.411812	4.44E-07	0.000295
4	*WFDC21P*	WAP four-disulfide core domain 21 pseudogene	3.014217	1.78E-11	3.03E-08
5	*CCL15*	Chemokine (C-C motif) ligand 15	2.713814	1.49E-16	8.90E-13
6	*SYNPO2*	Synaptopodin 2	2.366099	4.39E-06	0.001806
7	*CD1B*	CD1b molecule	2.346925	7.42E-05	0.017039
8	*IGFBP6*	Insulin-like growth factor binding protein 6	2.243955	4.46E-06	0.001806
9	*HCAR2*	Hydroxycarboxylic acid receptor 2	2.227582	6.71E-08	6.68E-05
10	*CXCL9*	Chemokine (C-X-C motif) ligand 9	2.186737	1.45E-05	0.005083
11	*PLTP*	Phospholipid transfer protein	2.176644	8.86E-08	8.14E-05
12	*MMP12*	Matrix metallopeptidase 12	2.136513	3.25E-05	0.009024
13	*SYNC*	Syncoilin intermediate filament protein	1.90768	0.000103	0.019723
14	*TNF*	TNF	1.823091	2.59E-06	0.001236
15	*HCAR3*	Hydroxycarboxylic acid receptor 3	1.818278	9.75E-05	0.019723
16	*COL1A1*	Collagen type I alpha 1	1.790855	4.54E-06	0.001806
17	*RAB30*	RAB30 member RAS oncogene family	1.729061	0.000104	0.019723
18	*IL32*	IL-32	1.717923	2.83E-06	0.001301
19	*BCL2L14*	BCL2-like 14 (apoptosis facilitator)	1.69596	2.40E-06	0.001195
20	*CCL17*	Chemokine (C-C motif) ligand 17	1.682015	0.000846	0.087868
21	*MMP19*	Matrix metallopeptidase 19	−1.50139	0.000943	0.09533
22	*RIN2*	Ras and Rab interactor 2	−1.50921	0.000283	0.041659
23	*DSC2*	Desmocollin 2	−1.59525	0.000109	0.020065
24	*CD14*	CD14 molecule	−1.63661	0.000815	0.086052
25	*GEM*	GTP binding protein overexpressed in skeletal muscle	−1.77586	3.82E-06	0.00169
26	*S100A8*	S100 calcium binding protein A8	−1.78739	0.000108	0.020065
27	*S100A9*	S100 calcium binding protein A9	−1.90406	2.92E-05	0.008483
28	*MMP10*	Matrix metallopeptidase 10	−1.93753	0.000251	0.039372
29	*TNFRSF21*	TNF receptor superfamily; member 21	−1.93923	4.99E-08	5.42E-05
30	*CDH23*	Cadherin-related 23	−2.01853	1.99E-07	0.000158
31	*MMP1*	Matrix metallopeptidase 1	−2.0201	0.000457	0.06061
31	*CCR1*	Complement component (3b/4b) receptor 1 (Knops blood group)	−2.02921	4.09E-08	4.88E-05
33	*SDC3*	Syndecan 3	−2.03712	1.71E-05	0.005516
34	*THRB*	Thyroid hormone receptor beta	−2.06934	2.99E-05	0.008483
35	*CCNA1*	Cyclin A1	−2.11411	1.82E-06	0.001034
36	*CD163*	CD163 molecule	−2.31895	0.000478	0.061497
37	*MMP8*	Matrix metallopeptidase 8	−2.32052	2.49E-05	0.007434
38	*VCAN*	Versican	−2.51837	2.20E-06	0.001142
39	*DYSF*	Dysferlin	−2.73433	6.34E-11	9.46E-08
40	*COL22A1*	Collagen; type XXII; alpha 1	−3.17478	4.57E-09	6.06E-06
41	*MUCL1*	Mucin-like 1	−3.36178	2.15E-06	0.001142
42	*FCMR*	Fc fragment of IgM receptor	−3.43339	2.58E-16	1.02E-12
43	*SCIN*	Scinderin	−4.48021	3.27E-14	8.25E-11

## Abbreviations

COL1A1: collagen type I alpha 1 chain
COL22A: collagen type XXII, alpha
DC: dendritic cell
DEGs: differentially expressed genes
DYSF: dysferlin
ECM: extracellular matrix
EGLN3: Egl-9 family hypoxia inducible factor 3 (PHD3)
FCMR: Fc fragment of IgM receptor
HCAR2: hydroxycarboxylic acid receptor 2
HCV: hepatitis C virus
IGFBP: insulin like growth factor binding protein
ISG: IFN-stimulated gene
MDM: monocyte-derived macrophages
MoDCs: monocyte-derived dendritic cells
MX1: MX dynamin-like GTPase 1
PLTP: phospholipid transfer protein
RELM-*β*: resistin-like beta
SCIN: scinderin
TIM-3: T-cell immunoglobulin and mucin-domain containing-3
VCAN: versican
WFDC21: WAP four-disulfide core domain 21.
