# Supplementary material for: Monocytes differentiated into macrophages and dendritic cells in the presence of human IFN‐λ3 or IFN‐λ4 show distinct phenotypes
Source: J Leukoc Biol. 2020 Nov 17;110(2):357–74. doi: 10.1002/JLB.3A0120-001RRR (PMC7611425; doi:10.1002/JLB.3A0120-001RRR)
Supplement: Supplementary file 1 — SUPPORTING INFORMATION [file JLB-110-357-s003.pdf]

Suppl. Fig. 1

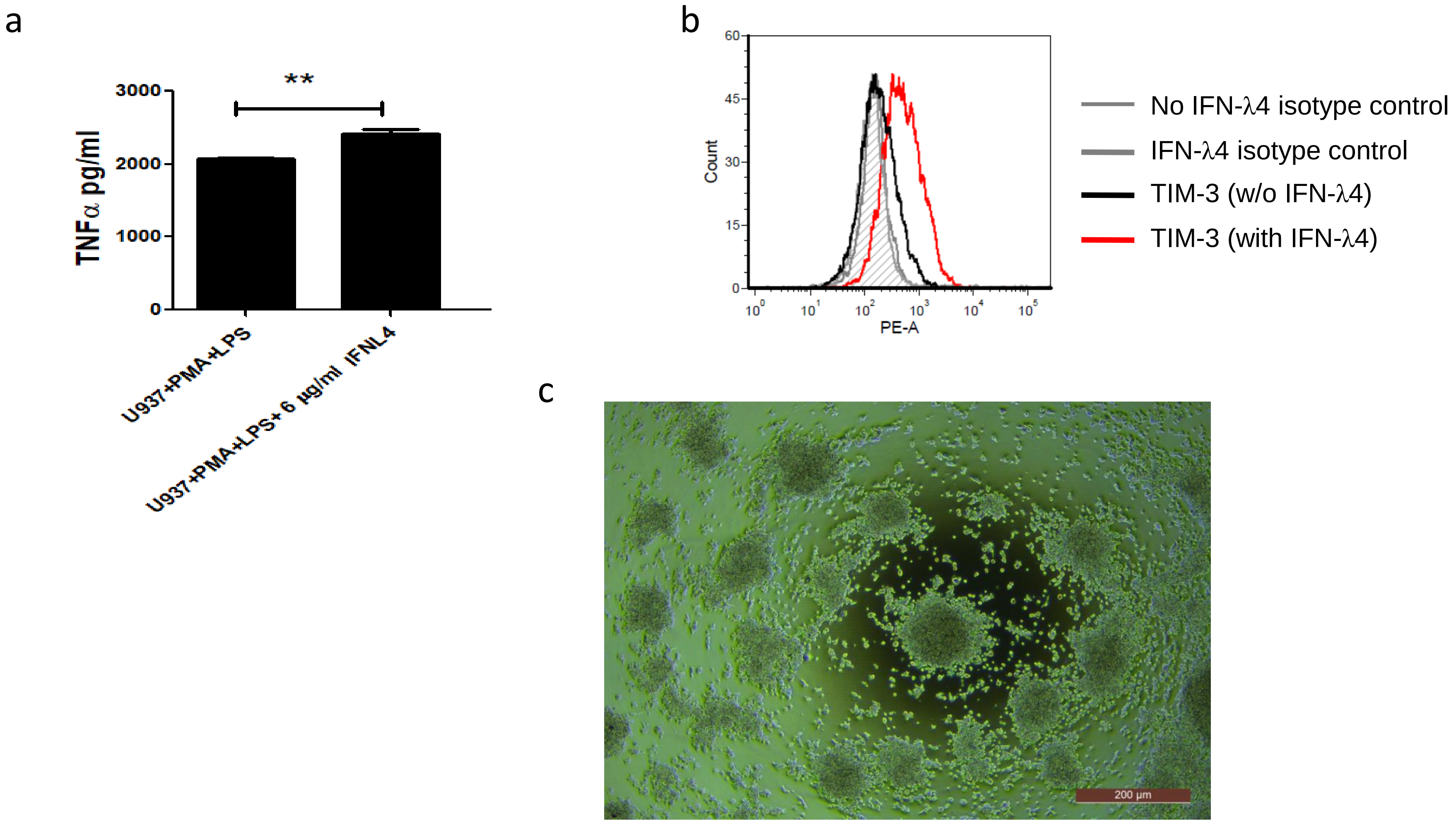

Suppl. Fig. 1: (a) TNF-α secretion was measured by enzyme-linked immunosorbent assay from cell-free supernatants of U937 cells treated with PMA in the absence or presence of IFN-λ4 for 48 h followed by incubation with LPS (pre-treatment strategy). **(b)** IFN-λ4 (6 µg/mL)-treated THP-1 macrophage-like cells activated with LPS show increased surface expression of TIM-3. **(c)** Microscopic image (amplification: ×10) of THP-1-derived M2 macrophage-like cells differentiated in the presence of IFN-λ4 (6 µg/mL).
