# Supplementary material for: Monocytes differentiated into macrophages and dendritic cells in the presence of human IFN‐λ3 or IFN‐λ4 show distinct phenotypes
Source: J Leukoc Biol. 2020 Nov 17;110(2):357–74. doi: 10.1002/JLB.3A0120-001RRR (PMC7611425; doi:10.1002/JLB.3A0120-001RRR)
Supplement: Supplementary file 2 — SUPPORTING INFORMATION [file JLB-110-357-s004.pdf]

Suppl. Fig. 2

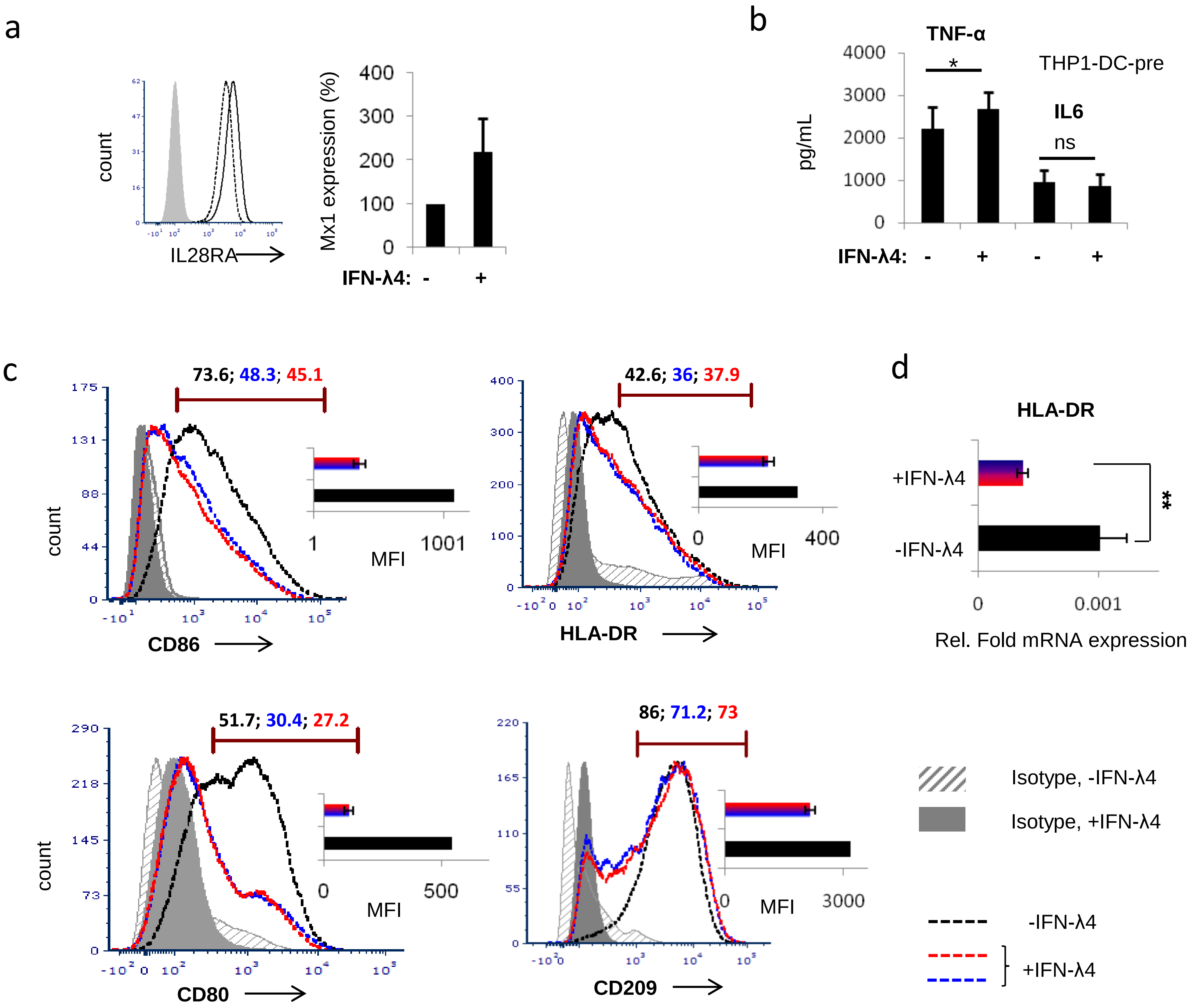

a
count
IL28RA
Mx1 expression (%)
400
300
200
100
0
IFN-λ4: - +
b
TNF-α
*
THP1-DC-pre
IL6
ns
pg/mL
4000
3000
2000
1000
0
IFN-λ4: - + - +
c
73.6; 48.3; 45.1
count
CD86
MFI
1
1001
42.6; 36; 37.9
HLA-DR
MFI
0
400
51.7; 30.4; 27.2
CD80
MFI
0
500
86; 71.2; 73
CD209
MFI
0
3000
d
HLA-DR
+IFN-λ4
-IFN-λ4
**
0
0.001
Rel. Fold mRNA expression
Isotype, -IFN-λ4
Isotype, +IFN-λ4
-IFN-λ4
+IFN-λ4

Suppl. Fig. 2: Characterization of the effect of IFN-λ4 on THP-1-derived dendritic cells (DCs). **(a)** Expression of IL28RA in THP-1-derived DCs treated for 24 h (solid line) and 48 h (broken line) with GM-CSF and IL-4; Mx1 gene expression by quantitative polymerase chain reaction (qPCR) from DCs derived from THP-1 by GM-CSF and IL-4 treatment for 48 h, followed by treatment with 6 μg/mL of IFN-λ4 for additional 24 h. **(b)** TNF-α and IL-6 secretion was measured by enzyme-linked immunosorbent assay from cell free supernatant of DCs derived from THP-1 cells differentiated in the absence or presence of IFN-λ4 (pre-treatment strategy) followed by maturation with LPS. The data is showing the mean from 12 replicates of four independent experiments carried out with three biological replicates each time, and standard deviation is shown by error bars. **(c)** Histogram of CD86, HLA-DR, CD80, and CD209 positive cells as determined by flow cytometry in DCs derived from THP-1 cells in the absence or presence of IFN-λ4 followed by maturation with LPS. Inset bar graph shows the median fluorescence intensity (MFI) and the numbers above the gates show the % counts for the respectively colored histograms. **(d)** qPCR showing down-regulation of *HLA-DR* expression in IFN-λ4-treated THP-1-derived DCs.
