# Supplementary material for: Monocytes differentiated into macrophages and dendritic cells in the presence of human IFN‐λ3 or IFN‐λ4 show distinct phenotypes
Source: J Leukoc Biol. 2020 Nov 17;110(2):357–74. doi: 10.1002/JLB.3A0120-001RRR (PMC7611425; doi:10.1002/JLB.3A0120-001RRR)
Supplement: Supplementary file 3 — SUPPORTING INFORMATION [file JLB-110-357-s001.pdf]

Suppl. Fig. 3

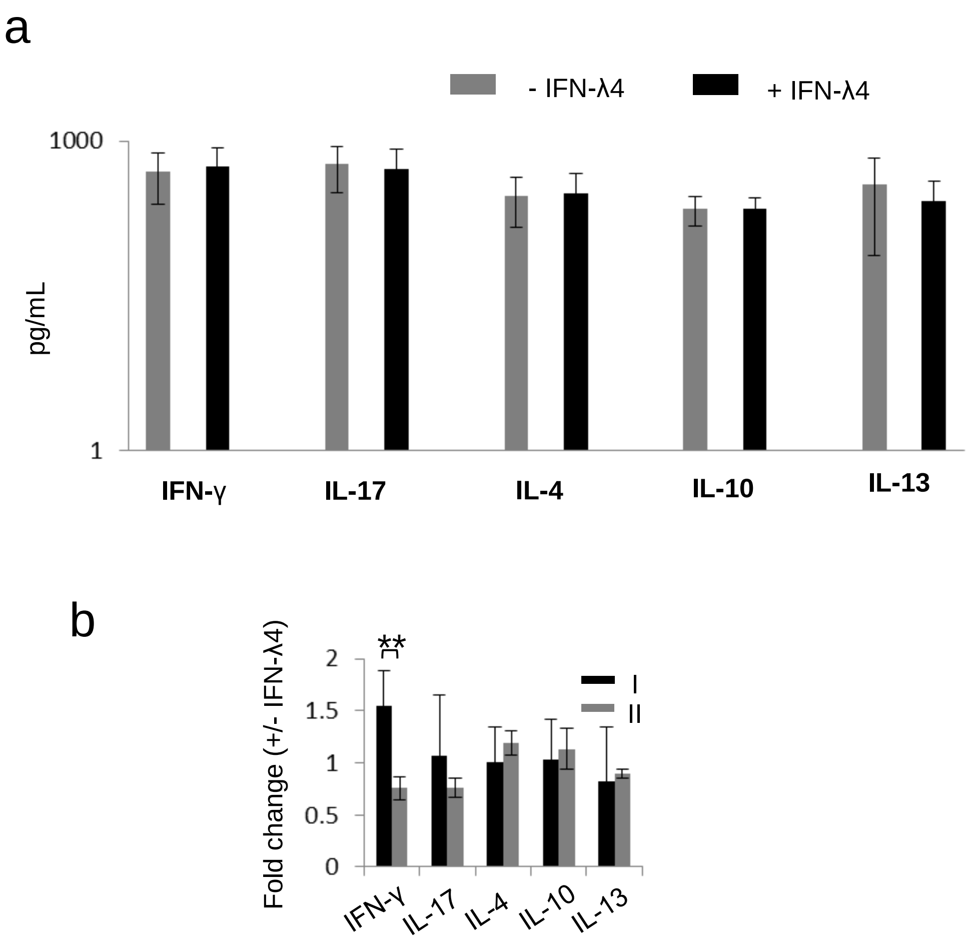

Suppl. Fig. 3: Co-culture of monocyte-derived dendritic cells (MoDCs) and T helper (Th) cells. MoDCs were derived from $CD14^+$ monocytes with GM-CSF and IL-4 in the presence or absence of 6 µg/mL IFN-λ4 (as described in method section). Cells were matured with LPS (1 µg/mL) and used to stimulate naïve $CD4^+$ T cells from eight unrelated donors. The secretion of IFN-γ, IL-17, IL-4, IL-10, and IL-13 were determined by enzyme-linked immunosorbent assay. **(a)** The bar graphs show the mean of secreted cytokines from all eight donor Th cells; secretion of cytokines in pg/mL and **(b)** fold change in cytokines secretion of IFN-λ4-treated cells vs mock-treated cells; the two groups (I and II) can be identified from within the eight donors (five in I and three in II) based on the secretion of IFN-γ.
