# Supplementary material for: Monocytes differentiated into macrophages and dendritic cells in the presence of human IFN‐λ3 or IFN‐λ4 show distinct phenotypes
Source: J Leukoc Biol. 2020 Nov 17;110(2):357–74. doi: 10.1002/JLB.3A0120-001RRR (PMC7611425; doi:10.1002/JLB.3A0120-001RRR)
Supplement: Supplementary file 4 — SUPPORTING INFORMATION [file JLB-110-357-s005.pdf]

Suppl. Fig. 4

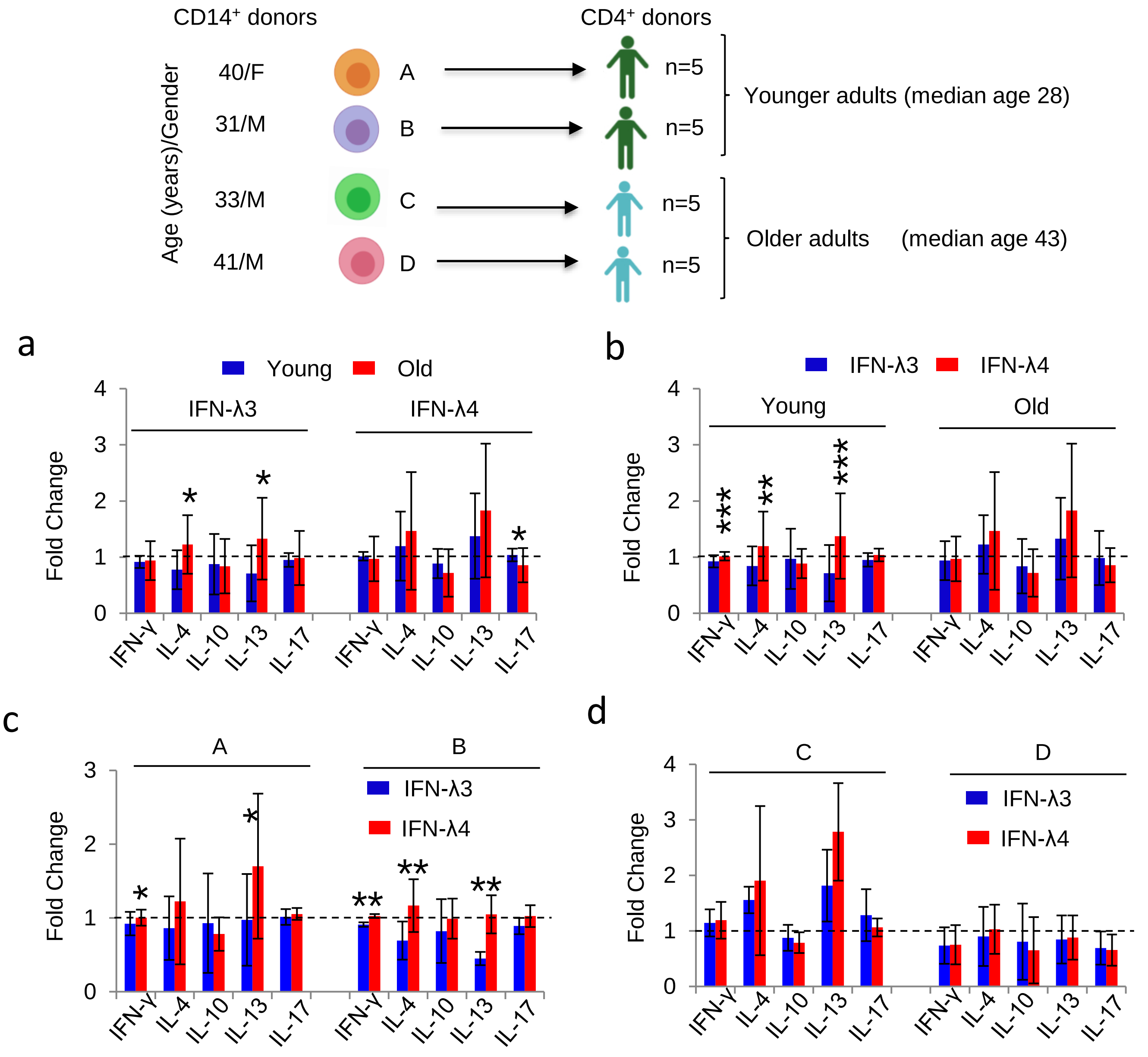

CD14⁺ donors
CD4⁺ donors
Age (years)/Gender
40/F
A
n=5
31/M
B
n=5
Younger adults (median age 28)
33/M
C
n=5
41/M
D
n=5
Older adults (median age 43)
a
Young
Old
IFN-λ3
IFN-λ4
Fold Change
IFN-γ
IL-4
IL-10
IL-13
IL-17
b
IFN-λ3
IFN-λ4
Young
Old
Fold Change
c
A
B
IFN-λ3
IFN-λ4
Fold Change
d
C
D
IFN-λ3
IFN-λ4
Fold Change

Suppl. Fig. 4: Monocyte-derived dendritic cells (MoDCs) differentiated in the presence of IFN-λ3 or IFN-λ4 show different fold changes of IFN-γ, IL-4, and IL-13 secretion from co-cultures involving $CD4^+$ T helper (Th) cells derived from younger donors. The schematic at the top shows the design of the experiment. All results shown are from enzyme-linked immunosorbent assay experiments. (**a**, **b**) Comparison of fold change in cytokine expression from co-cultures of $CD4^+$ cells from younger adult (n = 10) vs older adult (n = 10) donors with MoDCs differentiated from $CD14^+$ cell donors (A, B, C and D) in the presence of either IFN-λ3 or IFN-λ4. (**c**, **d**) Comparison of fold change in cytokine expression from co-cultures of $CD4^+$ cells and MoDCs derived from the four individual $CD14^+$ donors (A, B, C, and D) compared separately (n = 5) to detect individual effects of IFN-λ3 and IFN-λ4 on MoDCs. From **a-d** all the comparisons were made between the data points shown in blue vs red bars with one-tailed paired *t*-test; the bars depict average values and error bars depict standard deviation; *$p < 0.05$, **$p < 0.01$, ***$p < 0.001$.
