# Supplementary material for: Monocytes differentiated into macrophages and dendritic cells in the presence of human IFN‐λ3 or IFN‐λ4 show distinct phenotypes
Source: J Leukoc Biol. 2020 Nov 17;110(2):357–74. doi: 10.1002/JLB.3A0120-001RRR (PMC7611425; doi:10.1002/JLB.3A0120-001RRR)
Supplement: Supplementary file 5 — SUPPORTING INFORMATION [file JLB-110-357-s002.pdf]

Suppl. Fig. 5

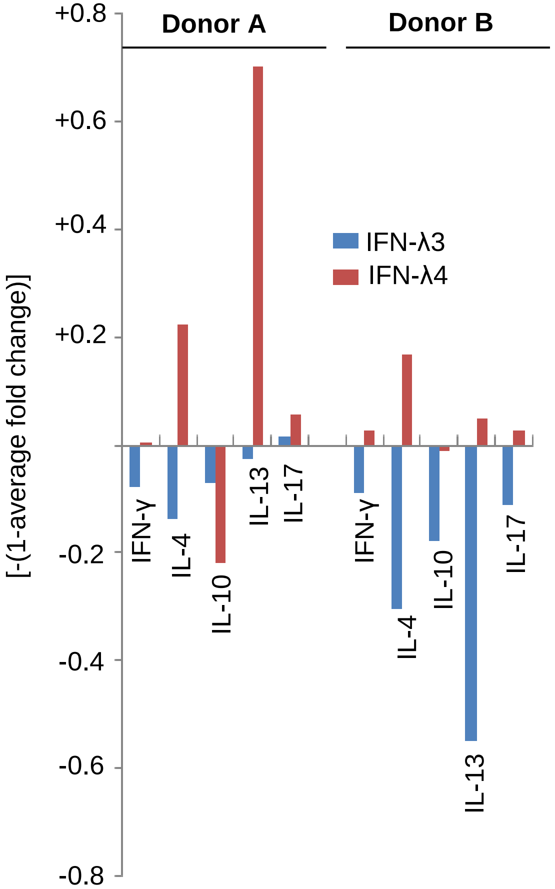

Suppl. Fig. 5: Monocyte-derived dendritic cells (MoDCs) differentiated in co-cultures in the presence of IFN-λ3 show an overall downregulation of cytokines, whereas those differentiated in presence of IFN-λ4 showed upregulated IL-4 and IL-13. The average fold changes for the respective cytokines from co-cultures involving MoDCs derived from $CD14^+$ donor A or B were subtracted from (+1) and multiplied by (-1) to obtain the values plotted on the Y-axis.
