# Supplementary material for: Monocytes differentiated into macrophages and dendritic cells in the presence of human IFN‐λ3 or IFN‐λ4 show distinct phenotypes
Source: J Leukoc Biol. 2020 Nov 17;110(2):357–74. doi: 10.1002/JLB.3A0120-001RRR (PMC7611425; doi:10.1002/JLB.3A0120-001RRR)
Supplement: Supplementary file 6 — SUPPORTING INFORMATION [file JLB-110-357-s006.docx]

**Suppl. Table 1:** **List of primers used in this study**

| **Gene** | **Forward primer sequence** | **Reverse primer sequence** |
| --- | --- | --- |
| **M1 and M2-MDM marker genes that showed significant changes in qPCR** | | |
| *CD80* | 5’-GGCCCGAGTACAAGAACCG-3’ | 5’-TCGTATGTGCCCTCGTCAGAT-3’ |
| *CD86* | 5’-CTGCTCATCTATACACGGTTACC-3’ | 5’-GGAAACGTCGTACAGTTCTGTG-3’ |
| *HLA-DR* | 5’-AGTCCCTGTGCTAGGATTTTTCA-3’ | 5’-ACATAAACTCGCCTGATTGGTC-3’ |
| *TIM-3* | 5’-CTGCTGCTACTACTTACAAGGTC-3’ | 5’-GCAGGGCAGATAGGCATTCT-3’ |
| *CXCL-13* | 5’-GCTTGAGGTGTAGATGTGTCC-3’ | 5’-CCCACGGGGCAAGATTTGAA-3’ |
| *IL-10* | 5’-GATGCCTTCAGCAGAGTGAA-3’ | 5’-CCCAGGTAACCCTTAAAGTCC-3’ |
| *RELM-β* | 5’-GTGGTTCGTGGGATGTTCAG-3’ | 5’-GGGACCCTGGTTTCATTACTG-3’ |
| *ICAM* | 5’-ATGCCCAGACATCTGTGTCC-3’ | 5’-GGGGTCTCTATGCCCAACAA-3’ |
| *VCAM* | 5’-GGGAAGATGGTCGTGATCCTT-3’ | 5’-TCTGGGGTGGTCTCGATTTTA-3’ |
| **M1 and M2-MDM marker genes that did not show significant changes in qPCR results in Fig. 3** | | |
| *CXCL10* | 5’-AGGAACCTCCAGTCTCAGCA-3’ | 5’-ATTTTGCTCCCCTCTGGTTT-3’ |
| *iNOS* | 5’-TTCAGTATCACAACCTCAGCAAG-3’ | 5’-TGGACCTGCAAGTTAAAATCCC-3’ |
| *IL-6* | 5’-TAATGGGCATTCCTTCTTCT-3’ | 5’-TGTCCTAACGCTCATACTTTT-3’ |
| *CD-68* | 5’-CTTCTCTCATTCCCCTATGGACA-3’ | 5’-GAAGGACACATTGTACTCCACC-3’ |
| *CXCL-9* | 5’-CCAGTAGTGAGAAAGGGTCGC-3’ | 5’-AGGGCTTGGGGCAAATTGTT-3’ |
| *CD-209* | 5’-AATGGCTGGAACGACGACAAA-3’ | 5’-CAGGAGGCTGCGGACTTTTT-3’ |
| *CCL-1* | 5’-CTCATTTGCGGAGCAAGAGAT-3’ | 5’-GCCTCTGAACCCATCCAACTG-3’ |
| *ARG1* | 5’-TACTAGGAAGAAAGAAAAGGCCAATTC-3’ | 5’-GTAGCCCTGTTTTGTAGATTTCTTCTGT-3’ |
| *IL-12* | 5’-ACCAGGTGGAGTTCAAG-3’ | 5’-TGGCACAGTCTCACTGTTGA-3’ |
| *TGF-b* | 5’-TACCTGAACCCGTGTTGCTCTC-3’ | 5’-GTTGCTGAGGTATCGCCAGGAA-3’ |
| *CD163* | 5’-GCGGGAGAGTGGAAGTGAAAG-3’ | 5’-GTTACAAATCACAGAGACCGCT-3’ |
| *CCL-17* | 5’-GCAAAGCCTTGAGAGGTCTTGA-3’ | 5’-CGGTGGAGGTCCCAGGTAGT-3’ |
| *CD11-b* | 5’-GCCTTGACCTTATGTCATGGG-3’ | 5’-CCTGTGCTGTAGTCGCACT-3’ |
| *CD18* | 5’-TGCGTCCTCTCTCAGGAGTG-3’ | 5’-GGTCCATGATGTCGTCAGCC-3’ |
| **ISGs tested in the study** | | |
| *MX1* | 5’-GGACATCACTGCTCTCATGC-3’ | 5’-TTTATGGCCTTCTTGAAAATTG-3’ |
| *Viperin* | 5’-CTTTGTGCTGCCCCTTGAG-3’ | 5’-TCCATACCAGCTTCCTTAAGCAA-3’ |
| *ISG15* | 5’-TCCTGCTGGTGGTGGACAA-3’ | 5’-TTGTTATTCCTCACCAGGATGCT-3’ |
| *OAS1* | 5’-AGAAATACCCCAGCCAAATCTCT-3’ | 5’-TGAGGAGCCACCCTTTACCA-3’ |
| *IFITM* | 5’-CTGGGCTTCATAGCATTCGCCT-3’ | 5’-AGATGTTCAGGCACTTGGCGGT-3’ |
| **Internal Control** | | |
| *GAPDH* | 5’-GGGTGTGAACCATGAGAAGTA-3’ | 5’-GGTGCAGGAGGCATTGCT-3’ |
